# Phenylphenalenones protect banana plants from infection by *Mycosphaerella fijiensis* and are deactivated by metabolic conversion

**DOI:** 10.1111/pce.12630

**Published:** 2015-12-11

**Authors:** William Hidalgo, Jima N. Chandran, Riya C. Menezes, Felipe Otálvaro, Bernd Schneider

**Affiliations:** ^1^ Max‐Planck Institut für Chemische Ökologie, Beutenberg Campus Hans‐Knöll‐Strasse 8 Jena 07745 Germany; ^2^ Instituto de Química Universidad de Antioquia Calle 67# 53‐108 Medellín A.A. 1226 Colombia

**Keywords:** *Musa*, detoxification, metabolomics, sulfate conjugates

## Abstract

Phenylphenalenones, polycyclic aromatic natural products from some monocotyledonous plants, are known as phytoalexins in banana (*Musa* spp.). In this study, ^1^H nuclear magnetic resonance (NMR)‐based metabolomics along with liquid chromatography and mass spectrometry were used to explore the chemical responses of the susceptible ‘Williams’ and the resistant ‘Khai Thong Ruang’ *Musa* varieties to the ascomycete fungus *Mycosphaerella fijiensis*, the agent of the black leaf Sigatoka disease. Principal component analysis discriminated strongly between infected and non‐infected plant tissue, mainly because of specialized metabolism induced in response to the fungus. Phenylphenalenones are among the major induced compounds, and the resistance level of the plants was correlated with the progress of the disease. However, a virulent strain of *M. fijiensis* was able to overcome plant resistance by converting phenylphenalenones to sulfate conjugates. Here, we report the first metabolic detoxification of fungitoxic phenylphenalenones to evade the chemical defence of *Musa* plants.

## Abstract 2

Both the susceptible ‘Williams’ and the resistant ‘Khai Thong Ruang’ varieties of *Musa acuminata* produced enhanced levels of phenylphenalenones in response to the infection with the ascomycete fungus *Mycosphaerella fijiensis*, the agent of the Black Leaf Sigatoka Disease.
The resistant *Musa* variety ‘Khai Thong Ruang’ produced a larger number and higher concentrations of phenylphenalenones than the susceptible variety ‘Williams’.
The virulent *M. fijiensis* strain Ca10_13 was able to overcome the resistance of the Musa variety ‘Khai Thong Ruang’ by metabolic deactivation of phenylphenalenones to sulfate conjugates.

## Introduction

Bananas and plantains constitute the fourth most important staple fruit worldwide with a global production of more than 100 million metric tons in 2012; they rank first among all varieties of fruits produced (FAO 2015). The revenues from trading these fruits internationally represent an important economical income for several tropical and subtropical developing countries (FAO 2014). However, their production is often affected not only by herbivores such as *Radopholus similis*, which cause the burrowing nematode disease (Chabrier & Queneherve 2003), but also by several pathogens: *Ralstonia solanacearum* (Race 2) causes the Moko's disease (Sequeira 1998); *Fusarium oxysporum* f. sp. *cubense* causes Panama's disease (Ploetz 2006); and the Sigatoka disease complex of banana, involving three related ascomycetous fungi *Mycosphaerella musicola*, *M. eumusae* and *M. fijiensis.* Among these fungi, *M. fijiensis* causes the most detrimental disease in banana, black leaf streak disease (BLSD) (Arzanlou *et al*. 2007; Etebu & Young‐Harry 2011).

Cavendish bananas, belonging to the subgroup AAA, are among the most frequently consumed commercial banana crops because of their sweetness and testability and the nutritional value of the fruit. However, they are highly susceptible to BLSD. Although several efforts have been made to breed *Musa* hybrids resistant to pathogens, the banana fruit for each cultivar type still does not meet consumers' expectations (Heslop‐Harrison 2011; Kovacs *et al*. 2013; Perrier *et al*. 2011; Vishnevetsky *et al*. 2011). Therefore, chemical treatments through the application of fungicides (such as benomyl, propiconazole and azoxystrobin) remain the most effective method to control this disease. As a consequence of poor agricultural management practices, namely, the blanket application of fungicides to avoid reduction in crop yields by fungal infections, banana plants have developed widespread resistance to fungicides. As a result, such reinforced microbial resistance has brought enormous losses in the commercial banana trade (Etebu & Young‐Harry 2011; Martinez‐Bolanos *et al*. 2012; Ploetz 2000).

Understanding the physiological responses triggered in resistant *Musa* plants during their interaction with pathogens, especially with *M. fijiensis*, will allow scientists to search for new strategies that can lead towards the control of pests without damaging the environment. Thus, several biochemical traits in *Musa* have been recognized to play a role during the pathogenesis. Among these, strengthened physical barriers through lignin biosynthesis, along with induction of pathogenesis‐related proteins, hypersensitivity response and production of phytoalexins, constitute the basis for the broad resistance of *Musa* plants to pathogens and herbivores (Dhakshinamoorthy *et al*. 2014; Hölscher *et al*. 2014; Otálvaro *et al*. 2007; Torres *et al*. 2012; Vaganan *et al*. 2014; Wang *et al*. 2015). Phenylphenalenones, a group of polycyclic aromatic compounds found in Haemodoraceae, Pontederiaceae, Strelitziaceae and Musaceae plant families (Munde *et al*. 2013), act as phytoalexins in the *Musa* genus because they are induced either in the leaves or in the roots of *Musa* plants that are affected by biotic or abiotic factors. In addition, their strong antimicrobial and nematicidal properties have made them valuable to researchers attempting to explain the chemical basis for the resistance of some cultivars of *Musa* (Hölscher *et al*. 2014; Jitsaeng & Schneider 2010; Quiñones *et al*. 2000).

As some phenylphenalenones have recently been identified at the cellular level in the stomata of *Musa* leaves (Hölscher *et al*. 2015), this experimental system has become attractive for exploring the plant–pathogen interaction in more detail. Furthermore, metabolomic analyses that use nuclear magnetic resonance (NMR) and mass spectrometry (MS) have been demonstrated to be powerful tools for analysing host metabolic pathways that are triggered by pathogens (Ali *et al*. 2009; López‐Gresa *et al*. 2010; Parker *et al*. 2009). The research reported in this study aimed to elucidate and explore the metabolic changes behind the *Musa–M. fijiensis* interaction, which is correlated with host resistance and pathogen virulence. Thus, chemical responses from the susceptible ‘Williams’ (*Musa* spp. AAA group) (Carlier *et al*. 2000) and the resistant ‘Khai Thong Ruang’ (‘KTR’) (*Musa* spp. AAA Ibota group) varieties in the interaction with *M. fijiensis*, strain E22 ‘sensitive’ and strain Ca10_13 ‘tolerant’ to the fungicide propiconazole, were probed by using ^1^H NMR‐based metabolomics along with high‐performance liquid chromatography (HPLC)–diode array detector (DAD) analysis.

## Materials and Methods

### Plant material

*In vitro* plants of *Musa acuminata* var. ‘Williams’ (AAA, Cavendish subgroup) were provided by Universidad Católica de Oriente – Rio Negro (Antioquía, Colombia), and *in vitro* plants of *M. acuminata* var. ‘KTR’ (AAA, Ibota subgroup) were originally provided by the International Transit Centre (ITC), Bioversity International, Katholieke Universiteit Leuven, Belgium (Leuven, Belgium). Plants were propagated and cultured individually in medium B5Z according to the standard procedures (Banerjee & Delanghe 1985) and maintained at 26 °C under 12 h photoperiod conditions until they reached a height of 10–12 cm (4–5 weeks). Plants were further transferred to 10 cm diameter plant pots containing sterile soil of a mixture of Klasmann‐Ton‐Substrat (KTS), vermiculite 2–3 mm, sand 0.7–1.2 mm and perlite (in a ratio 2:1:1:2) with fertilizer Oscomote Exact 19‐9‐12 (333 mg L^−1^ soil), and each plant received a dose of Ferty 3 (0.1%; Planta Düngemittel GmbH, Regenstauf, Germany) every week. The plants were maintained in an incubation chamber, Percival Model AR‐95HILX (CLF Plant Climatics, Emersacker, Germany), at a temperature of 26/23 °C (day/night), 70% humidity and 12 h photoperiod for 1 month. Then the plants were transferred to 4 L plant pots under the same soil conditions and kept for 3 months before the infection experiments began.

### Fungal organisms

*Mycosphaerella fijiensis* strains voucher E22 and voucher Ca10_13 were provided by Dr Gert Kema from the Plant Research International (Wageningen, the Netherlands) and maintained in solid potato dextrose agar medium (PDA, emended with 100 *μ*g mL^−1^ ampicillin), at 25 °C under dark conditions for 15 d. They were further cryo‐preserved according with the International Network for the Improvement of Banana and Plantain (INIBAP) technical guidelines (Carlier *et al*. 2002) until the inoculum for the infection protocol and bioassays was prepared.

### Analytical equipment

One‐dimensional (1D) and two‐dimensional (2D) NMR experiments were carried out using a Bruker Avance 500 NMR spectrometer (Bruker BioSpin, Karlsruhe, Germany), operating frequency 500.13 MHz for ^1^H and 125.75 MHz for ^13^C. A triple resonance inverse (TCI) cryoprobe (5 mm) was used to measure spectra at 300 K. Tetramethyl silane (TMS) (0.05%) was used as an internal standard for referencing ^1^H and ^13^C NMR spectra unless otherwise indicated. One‐dimensional Nuclear Overhauser Effect Spectroscopy (NOESY) experiments were performed using a Bruker Avance III HD 700 NMR spectrometer (Bruker BioSpin, Karlsruhe, Germany), operating frequency 700 MHz for ^1^H. A TCI H‐C/N‐D 1.7 mm microcryoprobe was used to measure spectra at 298 K. NMR spectrometers were controlled with bruker topspin 2.1 and 3.2 software (Bruker BioSpin, Karlsruhe, Germany) for the 500 and 700 MHz instruments, respectively. Electrospray ionization mass spectra (ESIMS) and LC–ESIMS were recorded on a Bruker Esquire 3000 ion trap mass spectrometer (Bruker Daltonics, Bremen, Germany). High‐resolution ESIMS was recorded on ultra high performance liquid chromatography (UHPLC) system of the Ultimate 3000 series RSLC (Dionex, Sunnyvale, CA, USA) connected to an LTQ‐Orbitrap XL mass spectrometer (Thermo Fisher Scientific, Bremen, Germany). Analytical and semipreparative HPLC was performed on an Agilent series HP1100 (binary pump G1312A, autosampler G1367A, diode array detector G1315A; Agilent Technologies, Waldbronn, Germany). The ultraviolet (UV) spectra were recorded by the DAD during analytical HPLC. The semipreparative HPLC system was coupled with the fraction collector CHF 122SB ADVANTEC (Dublin, CA, USA).

### Metabolomics analysis

#### Fungal inoculum preparation

The standard protocol reported by Abadie *et al*. (2008) was used with slight modifications. Briefly, *M. fijiensis* strain E22 kept in glycerol (15%) at −80 °C was placed in PDA medium and incubated at 25 °C under dark conditions for 20 d. Afterwards, the fungal mycelium was cut off from the agar medium and transferred to Petri dishes with sterile V8 juice agar (containing 300 mL of vegetable juice V8, 3 g CaCO_3_, 20 g of agar and 700 mL water) and incubated for 15 d under 12 h photoperiod. Four plugs of fungal mycelium were cut off from the Petri dish and placed in a Falcon tube (50 mL) containing 25 mL of sterile water for further shaking in a vortex mixer for 2 min followed by ultrasound for 1 min. An aliquot of 2 mL was transferred to Petri dishes with sporulation V8 juice agar (containing 100 mL of vegetable juice V8, 0.2 g CaCO_3_, 20 g of agar and 900 mL water, pH adjusted at 6.0) and incubated at 25 °C under continuous cool white light conditions for 15 d. Sterile water (~2 mL × 3) was added to the sporulation culture, and conidia were removed by brushing the colonies' surface with a sterile camel's hair brush. The concentration of conidia was calculated by using a Neubauer chamber and adjusted to a final concentration of 1 × 10^4^ conidia mL^−1^ with sterile water (containing 0.1% of Tween 20) and used to further inoculate the *Musa* plants.

#### Artificial mock‐inoculation of *Musa* plants

Three‐month‐old *M. acuminata* plants of the susceptible variety ‘Williams’ and the resistant variety ‘KTR’ were used for the infection experiment. A volume of 2 mL of the fungal inoculum was spread on the left‐half side of the lower surface (abaxial side) of the second youngest unfolded leaf of each *Musa* plant using a camel's hair brush. The mock‐inoculated plants were kept at room temperature (~20 °C) for 2 h, while the microbial suspension was dried and then returned to the climate chamber under the conditions described in the preceding text. The relative humidity was initially set at 90% and after the first week of incubation was maintained at 80%. A sterile solution of 0.1% Tween 20 (without fungal inoculum) was spread to *Musa* plants of each variety and used as a control. Four biological replicates of both treated and control *Musa* plants of each variety were used for the study.

#### Harvesting plant tissue

The infected *Musa* plants of each variety were harvested at 35 d post inoculation (dpi) once the plants developed visual BLSD symptoms on the upper side of the leaf. Four sections of each infected plant were collected as follows (Fig. 2): (1) necrotic plant tissue ‘sample A’; (2) asymptomatic tissue from the treated half of the leaf ‘sample B’; (3) healthy tissue from the non‐treated half of the infected leaf ‘sample D’; and (4) non‐treated leaves from the treated plant ‘sample E’. Plants without fungal treatment were used as a control, ‘sample C’. Each specimen was cut off under visual inspection (using a binocular loupe), immediately frozen in liquid nitrogen and stored in a pre‐cooled sterile Eppendorf tube (2 mL lock‐safe Eppendorf tubes) until further lyophilized in a freeze drier for 72 h. Ceramic beads (0.7 mm in diameter) were added to each tube, and the plant material was ground by shaking it for 3 min in a paint shaker (Skandex SO‐10M; Fluid Management Europe, Sassenheim, The Netherlands). The samples were then stored in a desiccator until they were used for analysis (not longer than 48 h).

#### Sample preparation for NMR analysis

A protocol established by Kim *et al*. (2010) was adapted with some modifications. Briefly, 25 mg of dried and ground plant specimens were transferred to an Eppendorf tube to which was added 0.8 mL of MeOD‐*d*_4_ with 0.2 mL of KH_2_PO_4_ buffer in D_2_O (pH 6.0) containing 0.05% trimethylsilyl propanoic acid (TSP) as internal standard. The solution was vortexed (1 min) and sonicated (15 min) at room temperature followed by a centrifugation step at 20,000 × g for 15 min. The supernatant (600 *μ*L) was directly transferred to a 5 mm NMR tube for further analysis.

#### NMR data analysis

^1^H NMR spectra were recorded at 500 MHz using a standard pulse sequence with water suppression (PURGE), with 256 scans with 64k data points and a spectral width of 15 ppm using a 30° flip angle, a 4.43 s acquisition time and a recycle delay of 2.0 s. Fourier transformation was applied to cumulative free induction decay (FID) with exponential line broadening of 0.3 Hz. All spectra were manually phased, baseline‐corrected and calibrated to TSP (δ 0.0). The ^1^H NMR spectra were reduced to 1586 sequentially integrated regions (‘buckets’) of 0.005 ppm width, from δ 0.5–8.7, and the data were scaled to total intensity using amix version 3.9.11 software (Bruker BioSpin, Karlsruhe, Germany). Regions from δ 3.32 to 3.25 and δ 5.0 to 4.8 were excluded before the bucketing analysis, and the whole dataset was exported as a text file (.txt). Principal component analysis (PCA) of the data was performed with simca‐p version 13.0 software (UMETRICS Company, Umeå, Sweden), and eight PCs were processed to meet 96% of the explained variance. Pareto or unit variance scaling was applied to the data before PCA analysis.

### Analysis of inducible phenylphenalenones in *Musa* during pathogen attack

#### Phytochemical analysis of infected *Musa* plants

The crude extract from sample ‘A’ of the variety ‘KTR’ (42.1 mg) and variety ‘Williams’ (34.3 mg) 35 dpi with the fungus was used to isolate the major metabolites induced during the pathogenesis. Briefly, the crude extract was cleaned up by using solid‐phase extraction (SPE, Chromabond C18ec‐octadecyl modified silica, 500 mg absorbent weight, endcapped; Macherey‐Nagel, Düren, Germany) before analysis by HPLC. The methanolic extract after SPE was dried under a stream of nitrogen and redissolved in methanol to a final concentration of 10 mg mL^−1^. An aliquot of 5 *μ*L of methanolic solution was injected into semipreparative HPLC. Separation was achieved on a Hibar column RP‐18e (Purospher STAR, 250–4.6 mm, 5 *μ*m particle size) (Merck, Darmstadt, Germany). Water with 0.05% trifluoroacetic acid (TFA) (solvent A) and a mixture of methanol:acetonitrile (1:1) with 0.05% trifluoroacetic acid (TFA) (solvent B) were used as a mobile phase. The elution profile was from 0 min (0% B), 60 min (45% B), 90 min (65% B), 110 min (65% B), 115 min (80% B), 125 min (80% B), 130 min (100% B) to 140 min (100% B). The mobile phase flow rate was 1 mL min^−1^. Column temperature was maintained at 30 °C. A wavelength at 254 nm was used for monitoring the chromatographic traces (HPLC method A). Further purification was carried out on a LiChrospher 100 RP‐18 column (250 × 4 mm, 5 *μ*m particle size) (Merck, Darmstadt, Germany) with water (0.1% TFA) and acetonitrile (solvent B) as a mobile phase. Flow rate of 1 mL min^−1^ and a linear gradient from 0 min (35% B) to 40 min (100% B) was used.

The crude extract of sample ‘D’ (asymptomatic area of infected plant) of the variety ‘Williams’ (18.2 mg) was processed following the same procedure as described before. Separation of the major compound was achieved by semipreparative HPLC using a LiChrospher 100 RP‐18 column (250 × 4 mm, 5 *μ*m particle size). Water with 0.1% TFA (solvent A) and methanol (solvent B) were used as the mobile phase. The elution gradient was from 0 min (0% B), 12 min (45% B), 17 min (100% B) to 25 min (100% B). Column temperature was kept at 30 °C. The monitored wavelength was 254 nm. One‐dimensional and 2D NMR experiments were carried out for the metabolites isolated.

#### Infection, sample preparation and disease rating

*Musa* plants of both varieties (‘Williams’ and ‘KTR’) were raised and treated with the fungus according to the conditions of the infection protocol with the fungus as described in the preceding text with few modifications. In this case, 5 mL of the fungal inoculum was spread on the whole (abaxial) lower surface of the second youngest unfolded leaf of each *Musa* plant using a camel's hair brush. Three areas of 4 × 4 cm^2^ were randomly traced with a permanent marker (Multimark 1523 Permanent, Faber‐Castell, Stein, Germany) on the upper surface of the infected leaf for determining the severity of the fungal pathogen over time. The average number and area of the symptoms were evaluated on the delimited area at 25 and 50 dpi with the fungus in order to determine the progress of the disease. Conidia of *M. fijiensis* strains E22 and Ca10_13, differing in their tolerance to the fungicide propiconazole, were chosen as infective propagules for both *Musa* varieties in separate experiments. The infected plant tissue (sample A, Fig. 2) was collected and processed as described in the preceding text. The experimental design consisted of six biological replicates of each plant variety per time assessed. Three plants without fungal inoculation were used as control (5 mL of water with 0.1% Tween 20).

#### Quantification by HPLC‐DAD analysis

9‐Phenylphenalenone was synthesized according to the protocol reported by Otálvaro *et al.* (Otálvaro *et al*. 2004), extensively purified by chromatographic techniques (column chromatography, preparative thin‐layer chromatography, semipreparative HPLC) up to a purity >98% (UV 254 and 280 nm) and used as internal standard for quantification of phenylphenalenones. A total of 25 mg of the infected dried and ground tissue was transferred into 0.8 mL methanol with 0.2 mL phosphate buffer and 0.1 mL of a 9‐phenylphenalenone solution (final concentration: 0.05 mg mL^−1^) for extraction and cleaning procedures as described in the preceding text, except that an SPE cartridge of 200 mg was used instead of a 500 mg SPE cartridge. The final extract was resuspended in 1 mL of a mixture of methanol:acetonitrile (1:1) and passed through a filter (0.45 *μ*m); the final solution (5 *μ*L) was injected into the HPLC using method A. The average of the peak integral value of three injections from each sample was used for quantification. Calibration curves of the phenylphenalenones and structural analogues (compounds **1–5**, **7**, **9–13** and **15** isolated and in‐house synthetic standards, purity > 98% checked by LC–MS, UV 254–280 nm) were generated using six standard solutions of each compound in the range of 0.1–1.5 mm (each solution containing the internal standard at a concentration of 0.05 mg mL^−1^). An aliquot of 5 *μ*L of each standard solution was injected in triplicate into the HPLC using method A. The average area of the peak integral value of each concentration was calculated from the triplicate data, and a linear regression equation was obtained by plotting the peak areas (*y*) versus the injected amounts (*x*) of each standard compounds. The linearity for each compound was evaluated by the correlation coefficient (*r* ^2^ ≥ 0.9967). The lower limit of quantification (LOQ) was 0.013 nmol per injection (based on the signal/noise ratio of approximately 10:1 extracted from the HPLC trace at 254 nm). Compounds **6**, **8** and **14** (Table 1) were quantified based on the relative response factor of hydroxyanigorufone (**5**), because the UV absorption of this compound at 254 nm was very similar to compounds **6**, **8** and **14**. The relative response factor of hydroxyanigorufone (**5**) was 0.5, as calculated relative to the internal standard, 9‐phenylphenalenone.

**Table 1:** Phenylphenalenones isolated by HPLC and identified by 1D and 2D NMR spectroscopy and HRMS from ‘KTR’ leaves after infection with *M. fijiensis* strain E22

No.	*R*_t_ (min)	Compound	Chemical structure	Occurrence in *Musa*	
				‘Williams’	‘KTR’
**1**	74.77	2‐(4′‐Hydroxyphenyl)‐1,8‐naphthalic anhydride	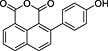	+	+
**2**	82.12	*trans*‐2,3‐Dihydro‐2,3‐dihydroxy‐9‐phenylphenalenone	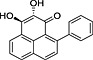	+	+
**3**	85.30	2‐Phenyl‐1,8‐naphthalic anhydride	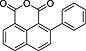	+	+
**4**	87.81	2‐Hydroxy‐4‐(4′‐hydroxyphenyl)‐1*H*‐phenalen‐1‐one (irenolone)	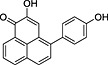	+	+
**5**	89.35	2‐Hydroxy‐9‐(4′‐hydroxyphenyl)‐1*H*‐phenalen‐1‐one (hydroxyanigorufone)	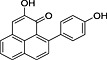	+	+
**6**	89.99	2‐Methoxy‐9‐(4′‐hydroxyphenyl)‐1*H*‐phenalen‐1‐one	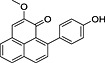	−	+
**7**	101.62	Methoxyanigorufone	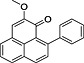	−	+
**8**	102.92	Dihydroxyanigorootin	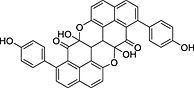	+	+
**9**	104.20	Anigorufone	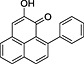	+	+
**10**	105.89	2‐Hydroxy‐9‐(4′‐methoxyphenyl)‐1*H*‐phenalen‐1‐one (4′‐*O*‐methylanigorufone)	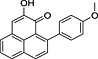	−	+
**11**	107.55	Isoanigorufone	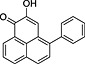	−	+
**12**	108.84	2‐Hydroxy‐4‐(4′‐methoxyphenyl)‐1*H*‐phenalen‐1‐one (4′‐*O*‐methylirenolone)	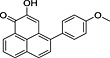	+	+
**13**	118.98	3,3′‐*bis*‐Hydroxyanigorufone	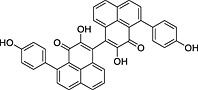	+	+
**14**	120.64	Hydroxyanigorootin	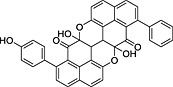	−	+
**15**	121.94	Anigorootin	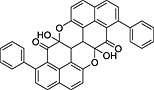	+	+

Occurrence of the metabolites is represented by (+) or absence/not detectable (−).

HPLC, high‐performance liquid chromatography; KTR, Khai Thong Ruang; NMR, nuclear magnetic resonance; 1D, one‐dimensional; 2D, two‐dimensional.

### Antifungal bioassay

A microtiter well method developed by Peláez *et al*. (2006) was used with some modifications (Hidalgo *et al*. 2009). Briefly, *M. fijiensis* strains (vouchers E22 and Ca10_13) grown for 15–17 d at 26 °C ± 1 in PDA (Fluka Analytical, Steinheim, Germany) were used for preparing the fungal inoculum. Mycelium of the culture was scraped with a smear loop and sterile water, and the dense suspension obtained was then fragmented in a vortex mixer with glass beads of 4 ± 0.3 mm diameter (Carl Roth GmbH, Karlsruhe, Germany) followed by filtration through four layers of sterile cheesecloth to obtain uniform myceliar fragments. The concentration of the inoculum was counted under a light microscope using a Neubauer haemocytometer (Marienfeld, Lauda‐Königshofen, Germany) and adjusted to 2 × 10^5^ mycelial fragments mL^−1^ with sterile water. Sterile microtiter plates 96 well‐F (Sarstedt AG & Co, Nümbrecht, Germany) were used for the biological incubation. Each well was filled with 50 *μ*L of Sabouraud broth (Fluka Analytical), 50 *μ*L of the fungal inoculum (described in the preceding text) and 50 *μ*L of the corresponding compound to be assessed. Concentrations ranging from 0.001 to 0.1 mg mL^−1^ of each compound were prepared in aqueous dimethyl sulfoxide (12% DMSO, BioReagent for molecular biology, ≥99.9%; Sigma‐Aldrich, Steinheim, Germany) and passed through a filter (0.1 *μ*m, hydrophilic polyethersulfone membrane sterile; PALL Life Sciences, Ann Arbor, MI, USA) before being administered to the incubation system. The culture medium (50 *μ*L; Sabouraud), 50 *μ*L of inoculum and 50 *μ*L of aqueous DMSO (12%, sterile) were used as a reference for growth control. Propiconazole (Fluka Analytical, 98.4%) was used as a positive control. The same procedure was applied for the negative control except that the inoculum solution was replaced by sterile water. The microplates were incubated under darkness or a photoperiod for 8 d (logarithmic growth phase of the fungi) at 25 °C in an incubation chamber. For the light‐controlled experiments, the plates were placed at a distance of 1 m and irradiated by two cool white light tubes (Sylvania Luxline Plus T8 36W/840, Havells Sylvania Großschirma, Germany) during 12 h d^−1^. Both experiments (darkness and photoperiod) were carried out simultaneously. The absorbance for the optical density (OD_595 nm_) was recorded by a spectrophotometer (TECAN Infinite M200 built‐in with multiple reads per well; TECAN, Crailsheim, Germany) at time 0 and 8 d after incubation in order to measure the mycelia growth. The experimental design consisted of three biological replicates (including three technical replicates). The half maximal inhibitory concentration (IC_50_) value was calculated by plotting the mycelial growth (data previously normalized to the reference control) against the logarithm of each concentration of the compound assessed.

### Phenylphenalenone metabolism

The antimicrobial activity of phenylphenalenones and derivatives depends on the chemical structure of the compound (Hidalgo *et al*. 2009; Otálvaro *et al*. 2007). In order to test whether the survival of *M. fijiensis* to the incubation with certain phenylphenalenones is in part due to the ability to metabolize or degrade them, an experimental approach was set up including several parameters to be analysed during *in vitro* interaction of the fungal pathogen and the phenylphenalenone. Thus, seven phenylphenalenone‐type compounds covering a range from weak to strong antifungal activity against *M. fijiensis* were selected for this analysis. The protocol described in the preceding text for the antifungal bioassay was used with some modifications. The fungal inoculum was prepared and adjusted to a final concentration of 3 × 10^5^ mycelial fragments mL^−1^ in identical conditions as has been described in the preceding text. Sabouraud broth (600 *μ*L) with 600 *μ*L of the fungal inoculum and 60 *μ*L of each concentration of the compound to be assessed was incubated in a sterile Safe‐Lock Eppendorf tube (2.0 mL; Eppendorf, Hamburg, Germany) containing five sterile ceramic beads (0.7 mm in diameter). Three solutions of each compound (0.21, 0.42 and 0.84 mg mL^−1^) were prepared in dimethyl sulfoxide (DMSO, BioReagent for molecular biology, ≥99.9%; Sigma‐Aldrich) to achieve a final concentration of 0.01, 0.02 and 0.04 mg mL^−1^ in the incubation system (maximum concentration of DMSO in the system: 4.7%). Sabouraud broth (600 *μ*L) with 600 *μ*L of the fungal inoculum and 60 *μ*L of DMSO (0.1 *μ*m filtered) was used as control growth. Similarly, 600 *μ*L of Sabouraud broth with 600 *μ*L of sterile water and 60 *μ*L of each compound solution (at each concentration) were prepared in order to ensure a comparable analysis with the treated samples. Ten replicates of control growth, reference compounds and treated samples were used for reproducibility purposes and additional analysis. The samples were maintained at 25 °C, shaken at 150 rpm under dark conditions and incubated for 8 d (logarithmic growth phase). Fungal biomass, soluble protein, ergosterol production and quantification of each phenylphenalenone either in the culture medium as well as in fungal mycelium were analysed as follows.

### Biomass quantification

After the microbial incubation, the fungal mycelium was completely removed from each tube and transferred to a new sterile Eppendorf tube. The fungal biomass was washed with 500 *μ*L (×3) phosphate‐buffered saline (sterile PBS, pH 7.4, 10% DMSO) and centrifuged at 20,000 × g for 10 min, and the supernatant was poured off and collected for further analysis. A final rinse was followed with 500 *μ*L of sterile water, and the mycelium was immediately frozen in liquid nitrogen and lyophilized in a freeze drier for 48 h. The biomass was left for 24 h in a desiccator before being weighed on a microbalance Mettler Toledo XP26 (0.001 mg readability; Giessen, Germany).

### Soluble protein quantification

The protein quantification was carried out by the Bradford method. Briefly, after 8 d of incubation with the fungus, 120 *μ*L of each culture medium (control growth, negative controls and treated samples) was placed in a sterile microtiter 96‐well plate and mixed with 120 *μ*L of Coomassie reagent (Coomassie Protein Assay Reagent; Thermo Scientific, Rockford, IL, USA) and then allowed to react for 15 min followed by reading the absorbance at 595 nm as indicated by the manufacturer. Three technical replicates of each sample were measured, and the average was used for quantification. The protein concentration was calculated from a calibration curve with bovine gamma globulin as standard.

### Ergosterol quantification

The fungal biomass obtained either from control as well as material treated with phenylphenalenones (*n* = 4) was ground in a paint shaker using stainless steel beads (1.4 mm in diameter; Carl Roth, Karlsruhe, Germany), and 0.7 mg was weighed, transferred to a Safe‐Lock Eppendorf tube (2.0 mL) and subjected to saponification with 1.5 mL of methanolic solution of KOH (0.1 m) followed by heating at 70 °C and shaking at 600 rpm for 10 min (Eppendorf Thermomixer Comfort 1.5 mL; Eppendorf, Hamburg, Germany). The reaction was left at room temperature, and then the tubes were centrifuged at 15,000 × g for 10 min and the supernatant transferred to a 4 mL glass vial. Distilled water (1 mL) was added to the methanolic solution along with 1 mL of diethyl ether for liquid–liquid extraction. The ether phase was poured off, and the extraction with diethyl ether was repeated twice. The organic phases were pooled and evaporated under a stream of nitrogen. The raw extract was resuspended in 60 *μ*L of CDCl_3_. ^1^H NMR spectra were recorded in a Bruker Avance 700 MHz spectrometer (Bruker Biospin, Karlsruhe, Germany) using a standard 1D NOESY pulse sequence with water suppression, with 512 scans with 32k data points. Other parameters were used as described in the preceding text for metabolomics analysis, and the chemical shifts were referenced to internal TMS (δ 0.0). Ergosterol was quantified using eretic2 software (Bruker Biospin, Karlsruhe, Germany) built‐in in topspin 3.2 (calibration method). Ergosterol (13.35 *μ* m; purity >98%) in CDCl_3_ was used as standard for the calibration procedure. The singlet signal at δ 0.63, which integrates for the protons of the methyl group attached to C‐13, was used for quantification. A minimal signal–noise ratio of 80:1 was calculated. A T_1_ relaxation time of 349.3 ms was determined for the signal at δ 0.63, and 5 × T_1_ was set up as a repetition time. Five standard solutions of ergosterol in the range 0.01–2.0 mm were prepared and evaluated for the accuracy of the molar quantification method. An area of at least five times the full width at half height (FWHH) of the ^1^H NMR signal was integrated and considered for the quantification. Absolute error below 2.34% was determined. This value is in agreement with validation methods using NMR spectroscopy (Pauli *et al*. 2005).

### Quantification of metabolized phenylphenalenones

To quantify phenylphenalenones, the culture medium was extracted with ethyl acetate (500 *μ*L × 5) by shaking for 10 min and centrifuged at 10,000 × g for 5 min. The pooled organic phase was evaporated under a stream of nitrogen gas. The extract was resuspended in 50 *μ*L of methanol, and an aliquot of 5 *μ*L of this solution was injected into HPLC. Separation was achieved in a Hibar column RP‐18e (Purospher STAR, 250–4.6 mm, 5 *μ*m particle size) using water (0.1% TFA, solvent A) and methanol (solvent B) as a mobile phase. A linear gradient was used from 0 min (40% B), 20 min (100% B) and 27 min (100% B). The monitor wavelength was set at 254 nm (HPLC method B). The analysis was carried out by triplicate injections of each sample and reference controls. A coefficient correlation *r* ^2^ ≥ 0.9997 was determined by plotting the peak area (average data) versus the concentration of each reference compound. The data were normalized by comparing the peak area of the compounds (average data) of the treated sample against the peak area (average data) of the reference compound. The LOQ calculated for the phenylphenalenones was used as described in the preceding text.

To quantify phenylphenalenones in the mycelium, fungal biomass was collected and lyophilized as described in the preceding text. Methanol (1 mL) was added to the fungal mycelium (0.7 mg), shaken at 650 rpm for 15 min at room temperature and centrifuged at 15,000 × g during 10 min at 4 °C. The extraction was repeated twice, and the organic extracts were pooled and evaporated under stream of nitrogen gas. The extract was resuspended in 50 *μ*L of methanol, and an aliquot of 5 *μ*L was injected into the HPLC system using method B. Triplicate analysis was conducted, and the data were normalized to the reference controls as was described in the preceding text.

### Analysis of phenylphenalenone metabolites

To investigate the products derived from the metabolism of phenylphenalenones by the fungus *M. fijiensis*, an up‐scaled incubation system with anigorufone (**9**) as reference compound was implemented. Thus, the fungal inoculum (3 × 10^5^ mycelial fragments mL^−1^) of the *M. fijiensis* strain Ca10_13 was prepared as described in the preceding text. A volume of 5 mL of inoculum was added to 50 mL of Sabouraud medium (sterile, incubation in Erlenmeyer flask volume 100 mL), and 2.38 mL of a solution of 1.5 m of anigorufone (prepared in 20% DMSO and passed through 0.1 *μ*m sterile filter) was added. A parallel experiment without any treatment was used to control fungal growth (2.38 mL of a solution 20% of sterile DMSO was added). The incubation was carried out at 25 °C, shaken at 150 rpm in a rotatory shaker under darkness for 12 d. Afterwards, the fungal biomass together with the culture medium was transferred to sterile Falcon tubes (50 mL) for centrifugation at 18,000 × g during 15 min. The supernatant was poured off to a new sterile Falcon tube (50 mL), and the mycelium was washed twice with 30 mL of PBS (pH 7.4) and rinsed with 30 mL sterile water (10% DMSO). A centrifugation step was used in each wash, and the supernatants were transferred to new sterile Falcon tube (volume 50 mL). Both fungal mycelium and the supernatants were frozen in liquid nitrogen, lyophilized for 72 h and then kept in a desiccator until further analysis. The lyophilized supernatants were resuspended in 5 mL of methanol and injected into LC–MS system using a LiChrospher 100 column RP‐18 (250–4 mm, 5 *μ*m particle size) with water (0.1% formic acid, solvent A) and methanol (0.1% formic acid, solvent B) as a mobile phase. A linear gradient was used from 0 min (40% B), 20 min (100% B) and 30 min (100% B). The fungal biomass [1.3 g dry weight (DW)] contained in a Falcon tube was ground using stainless steel beads (1.4 mm in diameter) and shaken in a paint shaker for 5 min. Methanol (10 mL × 3) was used for extraction by shaking for 10 min followed by centrifugation. The methanolic fraction was passed through a sterile membrane filter (0.45 *μ*m) and evaporated under a stream of nitrogen gas. The crude extract was resuspended in methanol (5 mg mL^−1^), and 5 *μ*L was injected into LC–MS system using the parameters described in the preceding text.

The methanolic extracts either from mycelium or from culture medium incubated with phenylphenalenones **5**, **7** and **9–12** (Table 1) were analysed by UHPLC–ESIMS. Phenylphenalenones were separated using an Acclaim C18 column (150 × 2.1 mm, 2.2 *μ*m; Dionex, Sunnyvale, CA, USA) with a flow rate of 300 *μ*L min^−1^ in a binary solvent system of water (solvent A) and acetonitrile (solvent B) (hypergrade for LC–MS; Merck, Darmstadt, Germany), both containing 0.1% (v/v) formic acid (eluent additive for LC–MS; Sigma‐Aldrich, Steinheim, Germany). Separation was accomplished using a linear gradient from 0 min (0% B), 15 min (100% B) and 20 min (100% B) and an equilibration time at 0% B for 5 min. ESI source parameters were set to 4 kV for spray voltage and 35 V for transfer capillary voltage at a capillary temperature of 275 °C. The samples were measured in positive and negative ion mode in the mass range of *m*/*z* 100 to 2000 using 30 000 m Δm^−1^ resolving power in the Orbitrap mass analyser. Data were interpreted using xcalibur software (Thermo Fisher Scientific, Waltham, MA, USA).

### Statistical analysis

Unless otherwise stated, statistical tests were carried out with sigma plot 12.0 (SYSTAT Software Inc., San Jose, CA, USA) using analysis of variance (anova). Leven's and Shapiro–Wilk tests were applied to determine error variance and normality of the data, respectively. Holm–Sidak *post hoc* test was used for pairwise or multiple comparison. Datasets that did not fulfil the assumptions for anova were natural log, decimal log, root square or rank transformed before analysis. A non‐parametric Kruskal–Wallis one‐way anova on ranks was applied for variables that did not meet the normality assumption.

## Results

### Differential responses of the interaction of *Musa–M. fijiensis* detected by principal component analysis

**Figure 1:**
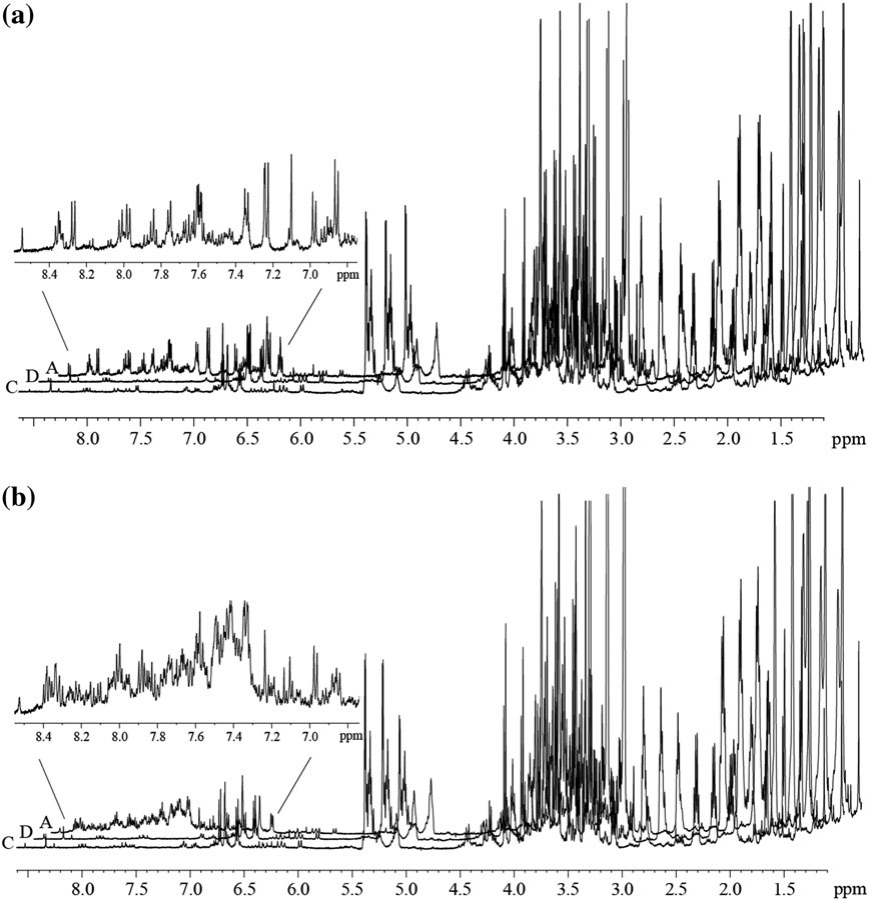
^1^H NMR spectra (500 MHz, MeOH‐*d*_4_) of crude extracts of *Musa* leaf tissues from varieties ‘Williams’ (a) and ‘KTR’ (b). Extracts were prepared from necrotic areas of the infected half (Fig. 2, A) and the non‐infected half (D) of a leaf treated with *M. fijiensis* strain E22, and a leaf of an untreated control plant (C) was taken 35 d after inoculation with fungal conidia. The extensions show the ^1^H NMR signals of induced phenolics in the necrotic leaf areas (A). Spectral intensities were scaled with the signal of trimethylsilyl propanoic acid (TSP), which was added as an internal standard.

**Figure 2:**
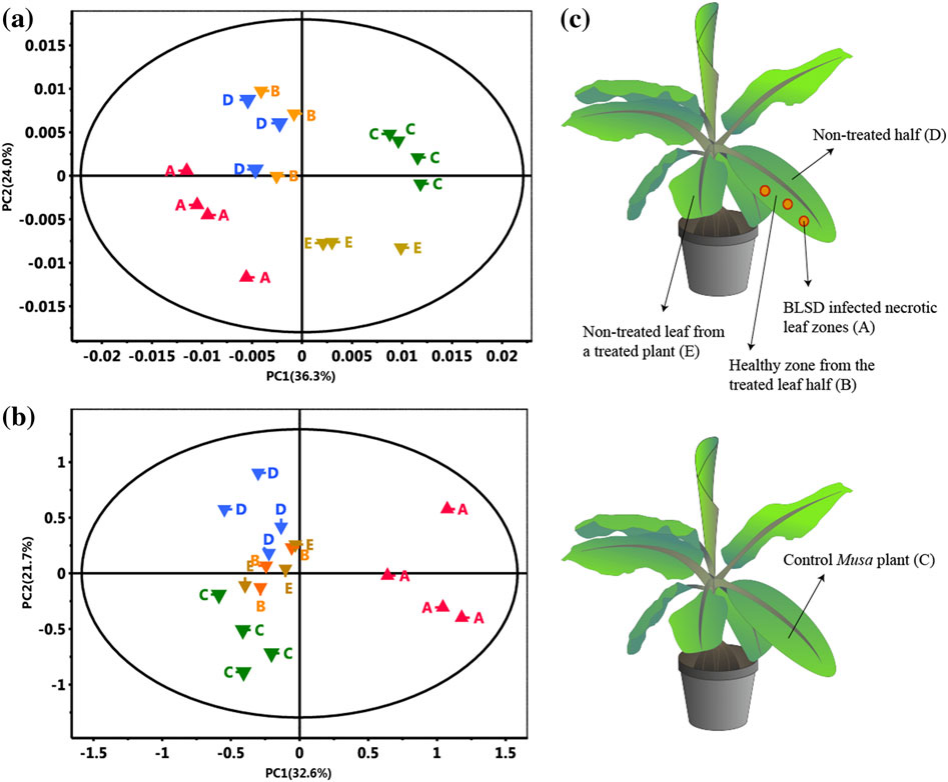
Score plots of PC1 versus PC2 of eight‐component principal component analysis (PCA) model of the region 0.5–8.7 ppm of the ^1^H NMR spectra (500 MHz) of samples A to E (0.005 ppm buckets). Regions 3.25–3.32 ppm and 4.8–5.0 ppm were excluded; data were normalized to total intensity of the spectrum. (a) ‘Williams’ variety (Pareto scaling was applied with *r* ^2^ = 0.903 of PCA model). (b) ‘Khai Thong Ruang’ (‘KTR’) variety (unit variance scaling was applied with *r* ^2^ = 0.854 of PCA model). (c) Sketch of the sampling procedure: A, black leaf streak disease (BLSD)‐infected necrotic leaf zones; B, healthy zones from the treated half of a leaf; C, control *Musa* plant; D, non‐treated half of a treated leaf; and E, non‐treated leaf from a treated plant.

In order to explore whether the susceptible *Musa* variety ‘Williams’ and the resistant variety ‘KTR’ respond differently to infection with the phytopathogenic ascomycete fungus *M. fijiensis*, *in vitro* plants of the two varieties were treated with conidia of the strain E22 as described in Section on Materials and Methods. The progress of the BLSD development was monitored by inspecting the leaf surface of the two *Musa* varieties using a binocular loupe. Initially, both the susceptible variety ‘Williams’ and the resistant variety ‘KTR’ developed similar symptoms at the site of treatment (Table S1). When BLSD symptoms of the treated plants, according to the classification by Fouré (1986), reached a dimension similar to stages 2 and 3, the necrotic zones (sample A) and healthy zones (B) of treated half of the leaves were probed (Fig. 2c). Simultaneously, further samples were taken from the non‐treated half of each treated leaf (D), non‐treated leaves of the treated plants (E) and untreated plants (control, C). When ^1^H NMR spectra of crude extracts of necrotic tissue (A) were compared with spectra of non‐necrotic tissue (D) from the same treated leaf and the control plant (C) (Fig. 1), signals of aromatic metabolites were more pronounced in the extracts of leaf areas where the fungal infection had taken place than in extracts of samples taken from ‘healthy zones’. This was observed both for ‘Williams’ and ‘KTR’. ^1^H NMR spectra of samples B and E (not shown in Fig. 1) closely resembled those of (C).

The ^1^H NMR spectra of all extracts are not much different in the regions of primary metabolites such as carbohydrates and amino acids (0.5–5.0 ppm, Fig. 1). However, the aromatic regions (6.0–9.0 ppm, Fig. 1) of the spectra exhibited not only largely enhanced signals obtained from the necrotic zones of infected leaves (A) but, compared with the spectra from extracts of non‐treated tissues, also a significantly increased number of signals. Thus, qualitative and quantitative gains in metabolic activity were observed in the infected zones of the plant and could be interpreted as a response to the pathogen attack. Therefore, it was thought that identification and quantification of the major metabolites produced while the BLSD ran its course would provide insight into the role and turnover of inducible natural products in this plant.

An NMR‐based metabolomic analysis was carried out to gain information about metabolic changes occurring in *Musa* plants under attack by the pathogen. The full ^1^H NMR spectra were used for understanding whether differences other than in the aromatic compounds exist between infected (A), non‐infected (B, D and E) and control (C) *Musa* plant samples (Fig. 2c). According to the score plots of PCA (Fig. 2a,b), the samples from BLSD‐infected necrotic leaf zones (A, red) were clearly discriminated from those of the control group (C, green) by the first principal component (Fig. 2a,b), mainly owing to aromatic compounds. Interestingly, the other non‐infected plant sections (asymptomatic areas B, non‐treated leaf areas D and E) obtained from plants treated with conidia of *M. fijiensis* E22 (Fig. 2c) appeared also separated in the PCA (blue, orange and gold) from the group of control *Musa* plants (green).

**Figure 3:**
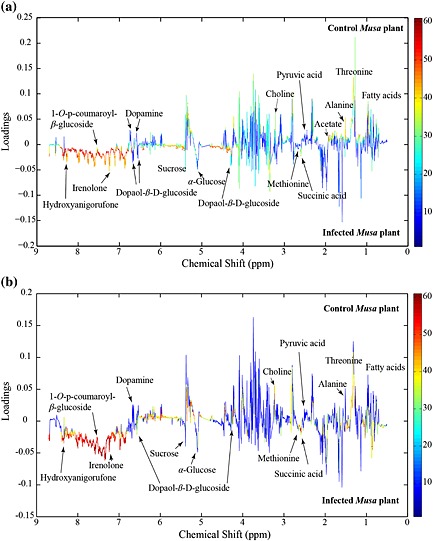
Loading plots for principal component analysis (PCA) model shown in Fig. 2a,b displaying the regions of the metabolites with strong influence (in red and orange) and the metabolites with less influence (light blue and dark blue) during the group discrimination observed in the PCA analysis. (a**)** ‘Williams’ and (b**)** ‘Khai Thong Ruang’ (‘KTR’) *Musa* varieties.

With the intention of detecting the signals in the spectra that contribute to the differences between samples, 1D and 2D NMR analysis was carried out along with comparisons with reference compounds and previously reported data (Franzyk *et al*. 2004; Huang *et al*. 2004; Kashiwada *et al*. 1988; Kim *et al*. 2010; Liang *et al*. 2006; Lu & Foo 2003; Najbjerg *et al*. 2011; Ramsay *et al*. 2014; Shimomura *et al*. 1987; Strack *et al*. 1989). This allowed the identification of the metabolites summarized in Table S2 (for chemical structures, see Fig. S1). Taking the information from the PCA, including consideration of the metabolites identified, the corresponding loading plots were constructed (Fig. 3). For both *Musa* varieties, the red‐orange color in the scale of the loading plots demonstrated that the phenolic metabolites, which are up‐regulated in infected tissue, permit the infected tissue to be discriminated from the non‐infected plant sections (Fig. 3). Interestingly, the level of the 1‐*O*‐((*E)‐p*‐coumaroyl)‐β‐d‐glucose was lower in infected tissue than in healthy tissue, perhaps because of consumption in specialized metabolism, which was triggered to produce phenylphenalenones (Table S2) such as irenolone and hydroxyanigorufone, already reported as phytoalexins of *Musa* plants (Luis *et al*. 1993, 1994, 1995). Further, among the identified metabolites, dopamine, a well‐known catecholamine in banana fruits (Kanazawa & Sakakibara 2000), and 2‐(3,4‐dihydroxyphenyl)ethyl β‐d‐glucopyranoside (common name dopaol β‐d‐glucoside), which is reported here for a first time as a metabolite from *Musa* plants, allowed the treated tissue to be discriminated from the untreated *Musa* tissue (for relevant resonances in the ^1^H NMR spectrum, see the region between 6.5 and 6.9 ppm in Fig. S2a,b).

A detailed analysis of ^1^H NMR spectra of all extracts of ‘Williams’ and ‘KTR’ showed that dopamine was present in infected leaves and in control plants. While control samples contained only traces of dopaol β‐d‐glucoside (Fig. S2c), its level almost equaled that of dopamine in the necrotic tissue of treated plants (Fig. S2a). In non‐infected zones from (B), (D) and (E) samples of both varieties, the level of the glucoside clearly exceeded the level of dopamine, which was detectable only in very low levels (Fig. S2b). This finding confirms the significance of dopaol β‐d‐glucoside in the differences observed by the PCA analysis.

According to the accumulation of dopaol β‐d‐glucoside in the different leaf areas of *Musa* plants treated with conidia of *M. fijiensis* but not in the control plant, this compound could be considered a phytoalexin; phytoalexins are produced in those areas of a plant where the pathogen has not been yet spread but which might already have received electrophysiological or chemical signals. Therefore, the antifungal property of dopaol β‐d‐glucoside was assayed against *M. fijiensis* in an *in vitro* experiment. However, antimicrobial inhibition was not observed, and the weak activity was comparable with that obtained for dopamine in the same assay (Fig. S3), ruling out the function as a phytoalexin. Thus, the biological function of this compound in the *Musa–M. fijiensis* pathosystem remains to be investigated, including its potential role in signalling.

In addition, the roots of infected and control plants were evaluated in both *Musa* varieties in order to check if specialized metabolites were observed in the roots as they were in the leaves. However, no differences between control and treated plants were found (data not shown), supporting the hypothesis that after *Musa* leaves are treated with the conidia of *M. fijiensis*, chemical defences are activated mainly in the above‐ground plant parts, especially at the infection site.

### Identification and quantification of induced defence metabolites in *Musa* after infection with *M. fijiensis*

#### Phytochemical analysis of infected *Musa* plants

Among the up‐regulated metabolites found by means of the previously described metabolomic approach, only a small number of phenolic compounds were identified (Table S2, entry 12–19). However, the many overlapping signals in the overcrowded aromatic region in the ^1^H NMR spectra (Fig. 1) suggested that more up‐regulated phenolics occur in these crude samples, especially in the ‘KTR’ variety, and made the identification of the signals difficult even using 2D NMR experiments. Therefore, a conventional phytochemical analysis of the infected leaves of both *Musa* cultivars showing BLSD symptoms (35 dpi) was carried out in order to identify the metabolites produced in response to the pathogen attack. HPLC‐guided isolation together with 1D and 2D NMR spectroscopy and high‐resolution mass spectrometry (HRMS) analysis allowed the identification of 15 phenylphenalenone‐type compounds or structural analogues in the resistant variety ‘KTR’ while 10 were found in the susceptible variety ‘Williams’ (Table 1). Among these compounds are irenolone (**4**) and hydroxyanigorufone (**5**), which have been identified in the metabolomic approach. Further compounds such as 2‐(4′‐hydroxyphenyl)‐naphthalic anhydride (**1**), methoxyanigorufone (**7**) and anigorufone (**9**) have been reported to be strongly toxic to fungi (Hirai *et al*. 1994; Kamo *et al*. 1998b; Lazzaro *et al*. 2004; Luis *et al*. 1995; Otálvaro *et al.* 2002a, 2002b, 2007). The ^1^H NMR spectra of the compounds from the infected *Musa* leaves matched those of authentic in‐house standards, and HRMS spectra were fully consistent with the suggested structures. All compounds, except compound **10**, are known natural products and have been identified from *Musa* plants (Kamo *et al*. 1998a, 1998b; Liu *et al*. 2014; Luis *et al*. 1993, 1994, 1995, 1999; Otálvaro *et al*. 2002a, 2002b). Unfortunately, some minor compounds remained unidentified because of the limited amount isolated and/or of insufficient chemical stability during the isolation procedure (data not shown). Their role in the plant defence therefore remains unclear.

#### Quantification of phenylphenalenone‐type compounds based on the progress of the disease

**Figure 4:**
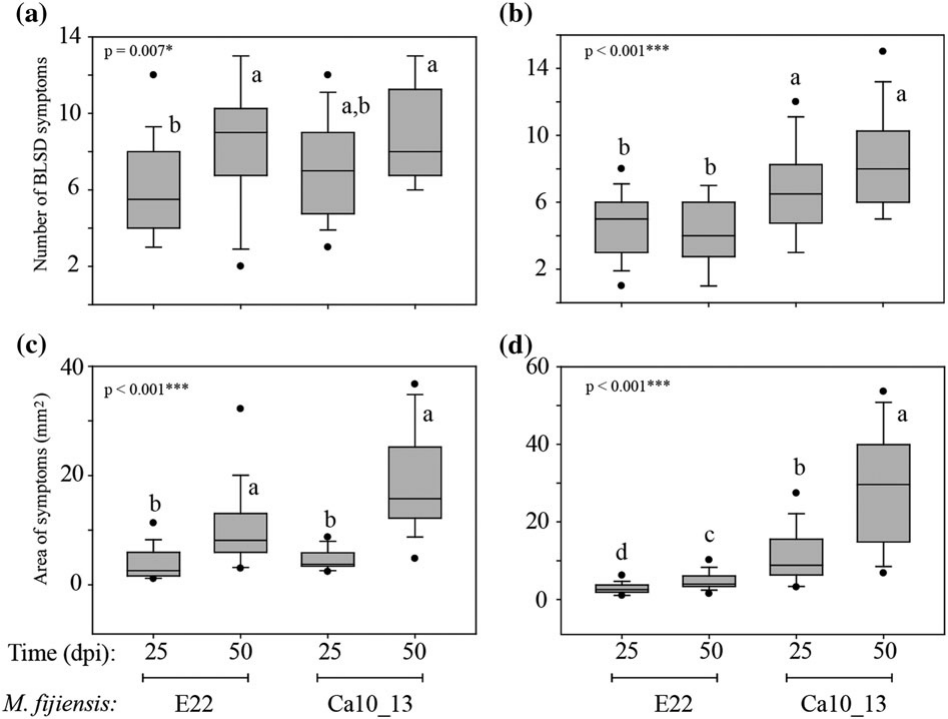
Number and area of black leaf streak disease (BLSD) symptoms determined for *Musa* ‘Williams’ (a,c) and ‘Khai Thong Ruang’ (‘KTR’) (b,d) varieties during the interaction with *M. fijiensis* strains E22 and Ca10_13. Letters a–d in the box plots indicate significant differences among the treatments. One‐way analysis of variance (anova), *P* < 0.001 = ***, *P* < 0.05 = *.

In order to study the hypothetical correlation between the virulence of *M. fijiensis* to *Musa* plants and the formation of phenylphenalenones, *in vitro* plants of ‘Williams’ and ‘KTR’ were treated in independent experiments with the fungal strains, E22 and Ca10_13, after which the symptoms were evaluated and the leaf tissues quantitatively analysed for the presence of phenylphenalenones. The two *M. fijiensis* strains were chosen according to their different tolerance (Ca10_13) or susceptibility (E22) to the fungicide propiconazole, and their conidia were used as infective propagules for exploring differential responses of susceptible and resistant *Musa* varieties. The virulence of the two strains was evaluated by visually inspecting the number and area of the lesions on the surface of the *Musa* leaves at different time points after infection (Fig. 4). At early stages of infection, the resistant variety ‘KTR’ exhibited some symptoms characterized by small red specks (~1 mm in diameter) on the lower surface of the leaf in response to each *M. fijiensis* strain, E22 and Ca10_13; these symptoms were stronger compared with symptoms observed in the susceptible ‘Williams’ variety (Table S1). This was evident by symptom‐free leaves in ‘Williams’ at 8 dpi. The further development of the BLSD symptoms is clearly different for the infection of the two *Musa* varieties treated with the strain E22 or Ca10_13. While the number and area of the lesions constantly increased in ‘Williams’, these symptoms remained almost unaltered on the leaves of ‘KTR’ after infection with E22 but increased after infection with Ca10_13, reaching severe damage after 50 dpi (Fig. 4, Table S1).

**Figure 5:**
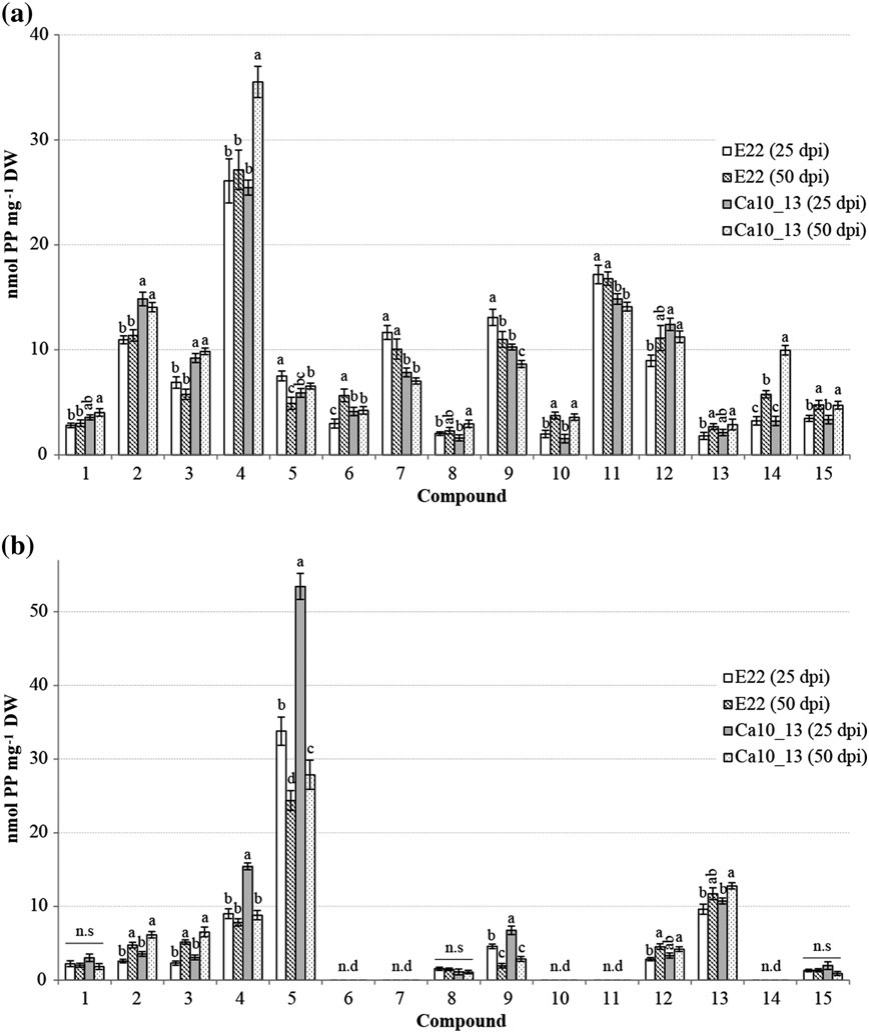
Quantification of the phenylphenalenones [nmol phenylphenalenone (PP) mg^−1^ dry weight (DW)] identified in ‘Khai Thong Ruang’ (‘KTR’) (a) and ‘Williams’ (b) varieties after infection with *M. fijiensis* strain E22 at 25 and 50 d post inoculation (dpi) and with *M. fijiensis* strain Ca10_13 at 25 and 50 dpi. Letters a–d indicate significant differences among treatments within the specified time (dpi) and compound [three‐way analysis of variance (anova), Holm–Sidak *post hoc* test: *P* < 0.05; n.s: not significant], n.d: not detectable. For chemical structures of compounds **1–15**, see Table 1.

After evaluating the BLSD symptoms, an HPLC–DAD approach was applied to quantify the inducible phenylphenalenone‐type compounds produced by the two *Musa* varieties ‘Williams’ and ‘KTR’ during their response to the pathogen. The concentration of phenylphenalenones was determined either in an early stage (25 dpi) or in a late stage (50 dpi) of the disease. The results for the ‘KTR’ variety (Fig. 5a) showed irenolone (**4**) as the major metabolite, with a concentration exceeding 25 nmol mg^−1^ DW. Metabolites **2**, **4**, **7**, **9**, **11** and **12** were produced in a concentration of >10 nmol mg^−1^ DW. All other compounds were produced in a concentration range between 1.5 and 9.5 nmol mg^−1^ DW.

A significant increase in the concentration of compounds **6**, **10** and **13–15** (*P* < 0.001) during the observed period of time from 25 to 50 dpi was found when the ‘KTR’ plants were treated with *M. fijiensis* strain E22. In contrast, compounds **5** (hydroxyanigorufone) and **9** (anigorufone) were significantly reduced in concentration between 25 and 50 dpi (*P* < 0.001) (Fig. 5a). When the plants were treated with *M. fijiensis* strain Ca10_13, mainly compounds **4** and **14** but also **8**, **10** and **15** were significantly induced at 50 dpi, while metabolite **9** again was found to be significantly reduced (*P* < 0.001).

The *Musa* ‘KTR’ variety reacted differently to the infection by each fungal strain. Overall, a significant increase in the production of compounds **2**, **3**, **4**, **12** and **14** were observed during the response against the strain Ca10_13, while compounds **6**, **7**, **9** and **11** were more induced by the infection with the strain E22 when compared with Ca10_13 (*P* < 0.001).

Like ‘KTR’, the variety ‘Williams’ also exhibited one dominant metabolite in addition to a number of medium‐concentrated and minor compounds. However, unlike ‘KTR’, which mainly produced irenolone (**4**), its positional isomer, hydroxyanigorufone (**5**) was the major compound in ‘Williams’ (25–52 nmol mg^–1^ DW) (Fig. 5b). Only two further compounds, **4** and **13**, also exceeded the concentration of 10 nmol mg^−1^ DW, while compounds **1–3**, **8**, **9**, **12** and **15** remained below that level and were produced in the range of 1.3–8.5 nmol mg^−1^ DW.

According to the statistical analysis, a significant increase in the time‐dependent induction of metabolites **2** and **3** were observed at 50 dpi, independently of the fungal strain used for the infection. Compounds **12** and **13** followed an induction pattern similar to that of **2** and **3** when the plant was treated with *M. fijiensis* strains E22 and Ca10_13, respectively. In contrast, the concentration of compounds **4**, **5** and **9** was dramatically reduced during the time BLSD developed, when the strain Ca10_13 was used (*P* < 0.001). A remarkable drop of the concentration of hydroxyanigorufone (**5**) from ~52 to ~27 nmol mg^−1^ DW was observed between 25 and 50 dpi. Similar results were found for compounds **5** and **9** after infection with strain E22 (Fig. 5b). The *de novo* biosynthesis of metabolites **1**, **8** and **15** remained almost unaffected both by the time point evaluated and by the fungal strain.

An overall overview of the total content of phenylphenalenones (and structural analogues) by the two *Musa* varieties was obtained by adding up the amount of each metabolite per milligram of plant dry material. Thus, both after inoculation with the strain E22 and the strain Ca10_13, the susceptible variety ‘Williams’ produced lower levels of phenylphenalenones compared with the resistant variety ‘KTR’ (Fig. S4). For treatment with E22, ~68 nmol mg^−1^ DW was found in ‘Williams’, and ~120 nmol mg^−1^ DW was found in ‘KTR’. No significant statistical differences in the total phenylphenalenone content were detected between plants at 25 dpi showing early BLSD symptoms and plants at 50 dpi showing stronger symptoms. However, large differences in total phenylphenalenone concentration between the early (25 dpi) and the late stage of the disease (50 dpi) were observed for both *Musa* varieties when infected with the *M. fijiensis* strain Ca10_13. ‘Williams’ and ‘KTR’ responded differently as the disease progressed. While the phenylphenalenone content in ‘Williams’ dropped significantly from ~100 nmol mg^−1^ DW at 25 dpi to ~70 nmol mg^−1^ DW at 50 dpi, the phenylphenalenone content in ‘KTR’ increased from ~120 to ~140 nmol mg^−1^ DW (Fig. S4).

### Antifungal bioassay of the major phenylphenalenones

**Figure 6:**
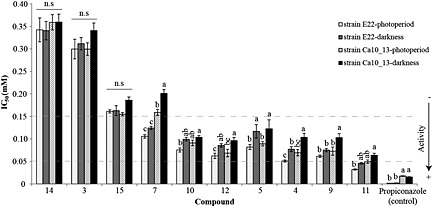
Half maximal inhibitory concentration (IC_50_) of the major inducible metabolites found in both *Musa* varieties during infection with *M. fijiensis*. Letters a–c indicate significant differences among treatments within the specified compound [two‐way analysis of variance (anova), Holm–Sidak *post hoc* test: *P* < 0.001; n.s: not significant]. For chemical structures of compounds **3–5**, **7**, **9–12**, **14** and **15**, see Table 1.

The major phenylphenalenone‐type compounds identified in both *Musa* plants were assayed for their antifungal properties against the two *M. fijiensis* strains E22 and Ca10_13 used in this study. The IC_50_ values were generated using the method reported by Peláez *et al.* (2006) with modifications as described in the Section on Materials and Methods and using propiconazole as a positive control. The results showed that both fungal strains were sensitive not only to the commercial fungicide propiconazole but, though to a different extent, also to most of the phenylphenalenone‐type compounds assessed. Among compounds **3–5**, **7**, **9–11**, **14** and **15** tested, only hydroxyanigorootin (**14**) and 2‐phenyl‐1,8‐naphthalic anhydride (**3**) did not display significant inhibitory activity against both *M. fijiensis* strains. Most of the IC_50_ values were found to be in the range between ~0.05 and ~0.15 mm (Fig. 6). Isoanigorufone (**11**), a 4‐phenylphenalenone found in the resistant variety ‘KTR’ but not in susceptible ‘Williams’, displayed antimycotic activity in IC_50‐E22_ = 0.032 ± 0.002 mm under light conditions. This value represented the best antifungal bioactivity among all the compounds tested. The antimicrobial activity of irenolone (**4**), hydroxyanigorufone (**5**) and 4′‐methoxyirenolone (**12**) against both *M. fijiensis* strains also increased significantly when the experiments were carried out under light‐controlled conditions (*P* < 0.05). The antifungal activity of anigorufone (**9**) and methoxyanigorufone (**7**) was also significantly light‐dependent but only in response to *M. fijiensis* Ca10_13 (*P* < 0.05) (Fig. 6). The results also showed that *M. fijiensis* Ca10_13 was not only tolerant to propiconazole but also to the phenylphenalenones assessed in this study. This became especially evident by comparing the growth of the strain E22 treated with anigorufone (**9**), irenolone (**4**) (under darkness only, *P* < 0.05) and methoxyanigorufone (**7**) (under darkness and photoperiod treatments, *P* < 0.001) with the growth of the strain Ca10_13 under the same conditions (Fig. 6).

### Metabolism of phenylphenalenones by the fungus

#### Motivation

The finding that, despite the induced production of antifungal phenylphenalenones, the BLSD symptoms caused by *M. fijiensis* strains E22 and Ca10_13 increased not only on the leaves of the susceptible *Musa* variety ‘Williams’ but also on the leaves of the resistant variety ‘KTR’ (for the case when the infection experiments were carried out with the strain Ca10_13) raised the question about the mechanism that enables the fungus to counteract the plant defence. The metabolism of defence chemicals is one of the most important ways the resistance of a plant is overcome by the pathogen (Morrissey & Osbourn 1999; Pedras & Ahiahonu 2005). The decreasing concentration of some of the most active antifungal compounds, especially anigorufone (**9**), during the progressing disease supported the suggestion that metabolic deactivation might be involved in the virulence mechanism of *M. fijiensis*. Therefore, selected phenylphenalenones were incubated under *in vitro* conditions with the fungus, and the mycelium and the fungal medium were screened for potential metabolites. Strain Ca10_13 was used in these experiments because its growth rate was inhibited to a lesser extent than the growth rate of strain E22 when incubated with phenylphenalenones (except 2‐phenyl‐1,8‐naphthalic anhydride (**3**) and hydroxyanigorufone (**5**), Fig. S5). Additionally, strain Ca10_13 was more virulent to banana plants compared with strain E22 (Fig. 4). Phenylphenalenones were applied at a concentration of 50% of the IC_50_. In addition to chemical analysis of phenylphenalenone‐derived metabolites, clues for the plant–fungus interaction such as the fungal biomass, the extracellular protein level and ergosterol concentration were determined.

#### Fungal biomass production during incubation with phenylphenalenones

Seven phenylphenalenone‐type compounds were incubated at three different concentrations (0.01, 0.02 and 0.04 mg mL^−1^) with the *M. fijiensis* strain Ca10_13. The results showed that anigorufone (**9**), 4′‐*O*‐methylirenolone (**12**) and 2‐phenyl‐1,8‐naphthalic anhydride (**3**) did not affect the production of biomass in comparison with the control (Fig. S6). However, incubation with isoanigorufone (**11**), hydroxyanigorufone (**5**) and 4′‐*O*‐methylanigorufone (**10**) resulted in a biomass production of up to ~80% in comparison with the control. The inhibition of the growth of fungal mycelium by compounds **5** and **10** was significantly dose‐dependent (Fig. S6). Interestingly, already the lowest applied concentration (0.01 mg mL^−1^) of methoxyanigorufone (**7**) significantly inhibited the biomass production to approximately 80% of the control. At concentrations of 0.02 and 0.04 mg mL^−1^, biomass production was dramatically reduced to approximately 30% of the control, demonstrating that the microorganism was enormously affected by this particular compound but not the other compounds.

#### Extracellular protein production

Extracellular microbial proteins can be involved in the degradation and deactivation of chemical defences of competing organisms (Morrissey & Osbourn 1999; Pedras & Ahiahonu 2005). Therefore, the production of soluble extracellular proteins was analysed in the culture medium in which the fungus was incubated with phenylphenalenone‐type compounds found in both *Musa* varieties. The aim of the analysis was to find out whether individual phenylphenalenones have a promoting or inhibiting effect on the protein production of the microorganism. The results showed clearly that isoanigorufone (**11**) and methoxyanigorufone (**7**) significantly induced the production of extracellular proteins in a dose‐dependent manner and caused pronounced increase when concentrations of 0.02 and 0.04 mg mL^−1^ were applied (Fig. S7). However, no significant differences in comparison with the control were found when the fungal culture was incubated with anigorufone (**9**), 4′‐*O*‐methylirenolone (**12**), 4′‐*O*‐methylanigorufone (**10**) and 2‐phenyl‐1,8‐naphthalic anhydride (**3**). Interestingly, hydroxyanigorufone (**5**) reduced the production of extracellular proteins to approximately 50% that of the control.

#### Quantification of ergosterol by ^1^H NMR analysis

Ergosterol is the major sterol of the fungal cell membranes and an important target molecule for many fungicides, which exert their mode of action through the inhibition of its biosynthesis (Debieu *et al*. 1998, 2013; Spotts & Cervantes 1986). In order to test whether phenylphenalenones affect ergosterol biosynthesis in *M. fijiensis*, a ^1^H NMR‐based quantification approach was used. Again, isoanigorufone (**11**) and methoxyanigorufone (**7**) were the metabolites that differ in their activity from the other compounds assessed. For **11**, a significant increase was observed when the compound was assessed at 0.01 and 0.02 mg mL^−1^, but this was not the case for the concentration of 0.04 mg mL^−1^, where the production of ergosterol was only slightly above the control level (Fig. S8). During the incubation with hydroxyanigorufone (**5**), an increase of the ergosterol concentration of 40% in comparison with that of the control was observed only when 0.02 mg mL^−1^ of the phenylphenalenone was applied. A similar increase of the ergosterol concentration was detected with 4′‐*O*‐methylirenolone (**12**), but no dose dependence was observed in this case. In contrast to treatment with compounds **5** and **12**, and especially with isoanigorufone (**11**), which all resulted in an enhanced ergosterol concentration, incubation of the fungus with methoxyanigorufone (**7**) drastically inhibited the production of ergosterol because it was not detectable at concentrations of 0.02 and 0.04 mg mL^−1^ Anigorufone (**9**) and 2‐phenyl‐1,8‐naphthalic anhydride (**3**) did not show significant differences in comparison with the control, and no dose dependence was detected.

#### Quantification of phenylphenalenones in *M. fijiensis* cultures

**Figure 7:**
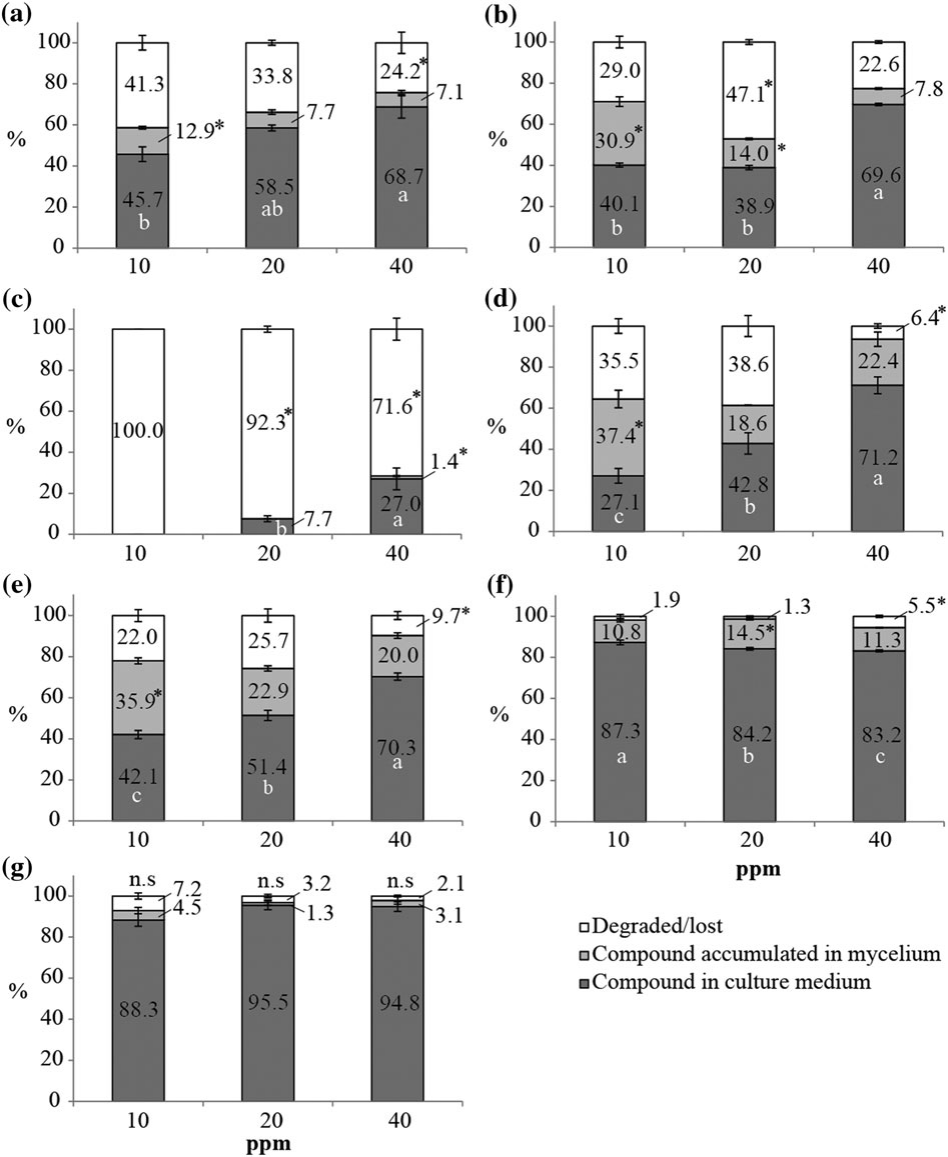
Quantification of phenylphenalenones after incubation of *M. fijiensis* strain Ca10_13 with different doses (0.01, 0.02 and 0.04 mg mL^−1^) of the specified compound. Data were normalized as a percentage based on the peak area generated by high‐performance liquid chromatography (HPLC)–diode array detector (DAD) analysis of the negative controls of each compound. Phenylphenalenone recovery from culture medium (dark‐grey bars), mycelium (light‐grey bars) and the unrecovered portions of phenylphenalenones were determined as the difference between the applied dose and the concentration determined in the medium and the mycelium after 8 d of incubation (white bars). (a**)** Isoanigorufone (**11**); (b) anigorufone (**9**); (c) hydroxyanigorufone (**5**); (d) 4′‐*O*‐methylirenolone (**12**); (e) 4′‐*O*‐methylanigorufone (**10**); (f) methoxyanigorufone (**7**); and (g) 2‐phenyl‐1,8‐naphthalic anhydride (**3**). Different letters or asterisks indicate significant differences among treatments (concentrations) of the compound analysed [one‐way analysis of variance (anova), Holm–Sidak *post hoc* test: *P* < 0.05; n.s: not significant].

The question of whether phenylphenalenone‐type compounds are taken up by the mycelial tissue or persist in the medium during incubation with the fungus was assessed by HPLC. Fungal cultures of strain Ca10_13 were incubated for 8 d with three different concentrations (0.01, 0.02 and 0.04 mg mL^−1^) of the corresponding phenylphenalenones, as described in the preceding text. Each phenylphenalenone was then quantitatively determined in the medium as well as in the mycelium. The results showed a very different distribution of the phenylphenalenones between the medium, the mycelium and a non‐recovered portion of the compounds (Fig. 7). A considerable portion of most compounds persisted in the medium. For compounds **9–12**, this portion was between 27 and 45% at the lowest concentration tested and increased up to approximately 70 to 80% at the highest concentration. Simultaneously, a certain percentage of compounds **9–12** accumulated in the mycelium biomass and showed a tendency to decline at higher doses of the applied compounds. According to the data shown in Fig. 7, the portions of compounds **9–12** detected in the medium and in the fungal mycelium do not sum up to 100%: an essential portion was missing, exceeding 40% in some cases. Again, the non‐recovered portion tends to decline with increasing doses of the applied compounds. However, generalizing with regard to the data is problematic, and an individualized consideration of the distribution pattern of most compounds seems to be reasonable, at least in the case of hydroxyanigorufone (**5**) and compounds **3** and **7**. Remarkably, compound **5** was no longer detectable after incubating a dose of 0.01 mg mL^−1^ with *M. fijiensis* strain Ca10_13, and even more than 70% of the highest applied dose of 0.04 mg mL^−1^ remained unrecovered. Unlike all other phenylphenalenones applied, methoxyanigorufone (**7**) and 2‐phenyl‐1,8‐naphthalic anhydride (**3**) were taken up by the mycelium only to a relatively small (~10% for **7**) or even negligible extent (**3**); that is, they remained mostly unchanged in the medium. For compound **3**, this finding coincided with its inactivity on the biomass production of *M. fijiensis* strain Ca10_13 as shown in Fig. S6.

#### Metabolism of phenylphenalenones by the fungus

The extensive (compounds **9–12**) or even complete (compound **5** at 0.01 mg mL^−1^) disappearance of phenylphenalenones (Fig. 7) during incubation with the fungus could be explained by metabolic processes. Therefore, in the next step of this study, the mycelium and the culture medium were analysed for potential metabolites of phenylphenalenones. In an up‐scaled incubation experiment with *M. fijiensis* strain Ca10_13, synthetic anigorufone (**9**) was used as a model compound to study the metabolism. Except for the parent compound **9**, no phenylphenalenone‐related compound was detected in the liquid culture medium, and the HPLC trace of the medium was identical with that of the control sample (data not shown). However, an additional peak, which was not present in the control sample, emerged in the UV and selected ion chromatograms (LC–MS, positive ion mode) of the methanolic mycelium extract (Fig. S9). The mass spectrum of this peak (*R*_t_ = 7.95 min) displayed a molecular ion of *m/z* 353.2 [M + H]^+^ and a fragment ion of *m/z* 273.2, suggesting a metabolite of compound **9**. HRMS analysis confirmed the fragment ion as anigorufone (**9**) ([M + H]^+^ found: *m/z* 273.0908, calc.: *m/z* 273.0916), and the molecular ion peak *m/z* 353.0475 ([M + H]^+^) was consistent with a sulfated metabolite of **9** ([M + H]^+^ calc.: *m/z* 353.0484). Because the hydroxyl group in position 2 of anigorufone (**9**) is the only place where a sulfate group could be attached to the molecule, the new metabolite must be anigorufone‐2‐sulfate (Fig. S9).

After anigorufone‐2‐sulfate was identified as a metabolite of anigorufone (**9**), the mycelia and extracts obtained after incubation of further phenylphenalenones were also analysed by UHPLC–HRMS for sulfate metabolites. While no such compounds were detected in the culture media, the mass spectra of the methanolic extracts of mycelia incubated with compounds **10** to **12** displayed not only peaks of the parent compounds but also characteristic peaks of corresponding sulfate conjugates (Table 2). Like anigorufone (**9**), compounds **10** to **12** have a single hydroxyl group at C‐2, and therefore, the sulfate unit of their conjugates must be attached to this particular position. A sulfate conjugate was also found for hydroxyanigorufone (**5**) (Table 2). As in the conjugates of compounds **10** to **12**, the position of the sulfate in the hydroxyanigorufone conjugate was arbitrarily assigned to the ortho‐hydroxyketone functionality, although two hydroxyl groups, 2‐OH and 4′‐OH, are available in this special case. No sulfate conjugate was produced during incubation of the methoxyanigorufone (**7**) with the fungus. This was not unexpected because no free hydroxyl group was available in this structure. Furthermore, as shown in Fig. 7, the uptake of compound **7** into the mycelia was very limited.

**Table 2:** Phenylphenalenone sulfates identified in methanolic extracts of mycelia after incubation of the parent phenylphenalenones with *M. fijiensis* Ca10_13

No.	Parent compound	Product	*R*_t_ (min)^a^	HRMS (*m/z*)	
				Calc.	Found
**5**	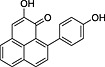	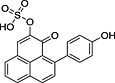 b	9.52	[M + H]^+^ = 369.0433	[M + H]^+^ = 369.0428
**7**	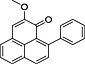	No metabolite detected	—	—	—
**9**	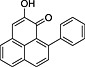	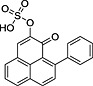	13.27	[M + H]^+^ = 353.0484	[M + H]^+^ = 353.0475
**10**	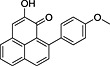	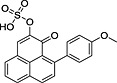	14.21	[M − H]^−^ = 381.0438	[M − H]^−^3 = 81.0435
**11**	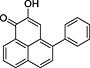	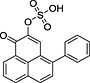	11.78	[M + H]^+^ = 353.0484	[M + H]^+^ = 353.0475
**12**	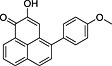	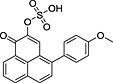	14.53	[M − H]^−^ = 381.0438	[M − H]^−^ = 381.0436

a Retention time of the phenylphenalenone sulfate [data from ultra‐high‐performance liquid chromatography–electrospray ionization mass spectra (UHPLC–ESIMS) analysis].

b The isomeric hydroxyanigorufone‐4′‐sulfate may be an alternative structure.

## Discussion

### Monitoring *Musa's* metabolic responses during *M. fijiensis* attack

^1^H NMR‐based metabolomics revealed similar biochemical profiles for both *Musa* varieties, ‘Williams’ (susceptible) and ‘KTR’ (resistant), in response to their interaction with the fungus *M. fijiensis*. Indeed, infection by this pathogen not only triggered the primary metabolism but also strongly affected the biosynthesis of specialized metabolites, especially aromatic compounds. These effects of infection were inferred from evaluating the group discrimination of each *Musa* plant part after the PCA (Fig. 2) and the corresponding loading plots (Fig. 3). While the levels of carbohydrates, represented mainly by glucose and sucrose, rose in the infected plant tissue, the levels of most amino acids (such as l‐alanine and l‐threonine) declined. l‐Methionine was an exception and tended to be more up‐regulated in the infected *Musa* plants than in the non‐treated plants. These findings confirm previous reports of plant–pathogen interactions that suggest that the translocation of sugars from the roots or other plant organs to the infected tissue may help counteract the metabolic imbalances caused by microbial invaders (Heil & Bostock 2002).

Carbohydrates are also involved in the biosynthesis of a broad spectrum of specialized metabolites and can mediate the gene expression of pathogenesis‐related proteins (Aliferis *et al*. 2014; Herbers *et al*. 1996; Herbers *et al*. 2000; Scharte *et al*. 2005). Gene expression demands a substantial pool of amino acids for the synthesis of proteins commonly associated with the defence against pathogens. For example, chitinases and glucanases have been reported to play a role in the interaction between *Musa* and *M. fijiensis* and in other plant pathosystems (Harish *et al*. 2009; Torres *et al*. 2012). The production of defence‐related proteins could explain the reduced content of amino acids found in the infected plant tissue of both *Musa* varieties. Surprisingly, l‐methionine, which was the only amino acid positively regulated during the infection, may be associated with enzymatic reactions that make up the methionine *S*‐methyltransferases involved in the *O*‐methylation of some phenylpropanoids and phenylphenalenone‐type compounds, as has been reported by Otálvaro *et al.* (2010).

An enhanced number of signals in the ^1^H NMR region from δ 6.0 to 9.0 were the most relevant features observed in the loading plots (Fig. 3) of both *Musa* varieties. Phenolic metabolites were more strongly up‐regulated in the infected local tissue of the resistant variety ‘KTR’ than in the infected local tissue of the susceptible variety ‘Williams’, thus providing the first hints that the two varieties respond differently to the infection with the pathogen.

Dopamine, an animal neurotransmitter also occurring in plants (Kulma & Szopa 2007; Li *et al*. 2015), is a catecholamine identified previously in banana fruits (Kanazawa & Sakakibara 2000). In our experiments, it was found in *Musa* leaves. We observed that it declined in the non‐treated areas of infected plants (Fig. 2, samples B and D). Simultaneously, a positive correlation was observed for dopaol‐β‐d‐glucoside, identified for the first time in healthy *Musa* plant tissue. Dopamine was thought to serve as a substrate in the biosynthesis of its glucoside, which then played a role as a phytoalexin. However, this assumption was ruled out because dopaol‐β‐d‐glucoside (and dopamine as well) did not show any antifungal properties against *M. fijiensis* (Fig. S3). Thus, the role of those metabolites in *Musa* plant defence remains to be studied.

A group of phenylpropanoid conjugates was identified for the first time as constituents of *Musa* plants (Table S2, entries 14–17). 1‐*O*‐((*E)‐p*‐Coumaroyl)‐β‐d‐glucose may function as a depot compound, which, after being hydrolyzed to *p*‐coumaric acid by a putative cell wall‐associated β‐glucosidase (Chong *et al*. 1999; Chong *et al*. 2002), could be used as a precursor for the biosynthesis of phenylphenalenone‐type compounds (Hölscher & Schneider 1995; Schmitt *et al*. 2000). This phenomenon could explain the reduced levels of such phenylpropanoids in the infected tissue. Moreover, phenylpropanoids are not only involved in the biosynthesis of phytoalexins of the phenylphenalenone‐type but also play a role in lignification processes in order to counteract the invading pathogen (Liang *et al*. 2006).

Irenolone (**4**) and hydroxyanigorufone (**5**), the two major phenylphenalenones identified from the metabolomic analysis (Table S2), were induced only in the infected plant tissue; along with other phenolic compounds, these major phenylphenalenones constituted the chemical basis for the *Musa* response to *M. fijiensis*. Unfortunately, because of the overlapping ^1^H NMR signals, metabolic changes of the other phenolics could be not observed in the loading plots (Fig. 3) but, rather, required a conventional phytochemical approach.

### Incompatible *Musa–M. fijiensis* interaction depends on the pathogen virulence

Phytochemical analysis of the infected *Musa* tissue led to the identification of 15 phenylphenalenone‐type compounds and structural analogues from the resistant variety ‘KTR’. A smaller number (10) of such metabolites appeared in the defence response of the susceptible variety ‘Williams’. With the exception of 2‐hydroxy‐9‐(4′‐methoxyphenyl)‐1*H*‐phenalen‐1‐one (**10**), all other compounds had been previously reported in different varieties of *Musa*, and their occurrence has been correlated with the resistance phenotype exhibited by some *Musa* varieties against pathogens and herbivores (Hölscher *et al*. 2014; Otálvaro *et al*. 2007). Nonetheless, there has been little quantitative analysis of phenylphenalenones as BLSD progresses, and resistant traits have not been studied with isolates of *M. fijiensis* that differ in their virulence. Therefore, two strains of *M. fijiensis*, E22 and Ca10_13, were used as inoculum. According to the method proposed by Cañas‐Gutierrez *et al.* (2009), the strain E22 was classified as ‘sensitive’ and Ca10_13 as ‘tolerant’ to the fungicide propiconazole. It is important to note that sensitivity to the fungicide propiconazole does not necessarily correlate with a high degree of virulence of the pathogen. However, fungicide tolerance could help the microorganism overcome the plant's chemical defence and/or synthetic fungicides.

For the variety ‘KTR’, an early response was observed by the appearance of small specks on the lower surface of the infected leaf 7–9 dpi with the fungus, regardless of which *M. fijiensis* strain was used; no symptoms were detectable in the variety ‘Williams’ up to 15–17 dpi (Table S1). This difference between varieties is not surprising because an early recognition of virulence effectors from the pathogen triggers the defence mechanism in *Musa* (as they do in other plant–pathogen systems), conferring resistance on plants that are attacked (Hoss *et al*. 2000; Torres *et al*. 2012).

The resistance conferred on *Musa* ‘KTR’ in its interaction with *M. fijiensis* strain E22 was confirmed by the slow progress of BLSD determined at the two points of time. In contrast, *M. fijiensis* Ca10_13 not only exhibited a higher degree of virulence than E22 but also was able to overcome the resistance of ‘KTR’ and cause severe foliar damages in the infected leaf (Fig. 4, Table S1). According to previous reports, the breakdown of the resistance in *Musa* caused by *M. fijiensis* has also been observed in other *Musa* varieties such as the variety ‘Yangambi Km5’, which is classified as highly resistant to this particular pathogen (Fullerton & Olsen 1995; Mouliom‐Pefoura 1999).

For the variety ‘Williams’, the necrotic symptoms of BLSD were larger than those observed for ‘KTR’ during the progress of the fungal infection; this difference was expected for a susceptible variety. Nonetheless, leaf damage was increased when *M. fijiensis* Ca10_13 was used as a pathogen, confirming the strong virulence of this pathogen.

Priming defence mechanisms in plants at early stages of infection, after the recognition of the effectors signals produced by a pathogen, are crucial in the incompatible plant–pathogen interactions (Conrath *et al*. 2006; Frost *et al*. 2008). As demonstrated for some resistant *Musa* varieties, the production of pathogenesis‐related proteins, lignification processes, hypersensitivity response and accumulation of phytoalexins must work in a concerted action to enable plants to block pathogenesis and herbivory (Harelimana *et al*. 1997; Hölscher *et al*. 2014; Otálvaro *et al.* 2002a, 2002b; Quiñones *et al*. 2000; Torres *et al*. 2012).

Here, the BLSD symptoms appearing on the leaves of the ‘KTR’ variety after infection with the strain Ca10_13 clearly demonstrated that resistance in this *Musa* variety could be overcome by a highly virulent pathogen. Analysis of the chemical composition of both *Musa* varieties during their responses to the two fungal strains showed that phenylphenalenone‐type compounds constituted the major induced metabolites both in ‘Williams’ and in ‘KTR’. The susceptible variety ‘Williams’ produced not only a smaller number but also lower levels of these metabolites (less than 10 nmol mg^−1^ DW) compared with ‘KTR’. Hydroxyanigorufone (**5**) appeared to be the exception: it was the most prominent compound synthesized in ‘Williams’ in response to the strain Ca10_13. Nevertheless, as the disease developed, the concentration of hydroxyanigorufone (**5**) and anigorufone (**9**) decreased, while that of other compounds (e.g. **1–4**) stayed constant or even slightly increased. The reduced levels of compounds **5** and **9** at 50 dpi could be due to an oxidative metabolic reaction and may result in the naphthalic anhydrides **1** and **3**, respectively. Reactive oxygen species like hydrogen peroxide, which have been reported to be produced in the infected tissue of *Musa* plants (Torres *et al*. 2012), could function as the oxidizing agent. In ‘Williams’, the susceptibility seemed to be associated with a late recognition of the pathogen, which then successfully colonizes the plant tissue. This is consistent with a relatively low total level of phenylphenalenones (Fig. S4), although hydroxyanigorufone (**5**), which is one of the active phenylphenalenones (Fig. 6), was overproduced in comparison with the other metabolites (Fig. 5b).

In contrast, the ‘KTR’ variety not only rapidly recognized the pathogen but also responded with a burst of phenylphenalenones, providing an enhanced chemical response to both fungal strains. This variety produced a number of phenylphenalenones in concentrations over 10 nmol mg^−1^ DW; irenolone (**4**) was the most abundant compound (>25 nmol mg^−1^ DW) (Fig. 5a). Compound **4** and its congeners seemed to have a strong inhibiting effect on the fungal colonization of plant tissue infected by the strain E22 but not on tissue infected by Ca10_13 (Fig. 5a). The response of the two *Musa* varieties to the two fungal strains E22 and Ca10_13 produced slight quantitative differences but no qualitative differences in the metabolic patterns (data not shown).

In ‘KTR’, the concentrations of compounds **2–4**, **12** and **14** increased significantly in response to Ca10_13 at 25 or 50 dpi but not in the response to the strain E22. Irenolone (**4**) was the metabolite with the highest response against Ca10_13 at 50 dpi in comparison with all other induced metabolites (Fig. 5a). This finding suggests that, at least from the chemical point of view, ‘KTR’ was able to distinguish between the virulence capacities of the fungal strains.

### Pathogen virulence associated with metabolism of phenylphenalenones

The breakdown in the resistance of ‘KTR’ caused by the virulent strain Ca10_13 encouraged us to explore whether some phenylphenalenones (e.g. compounds **3**, **9**, **11**, **12**), although up‐regulated in response to *M. fijiensis*, did not significantly inhibit the growth of this particular fungus *in planta*. The antifungal bioassay showed that phenylphenalenones were active against both fungal strains (Fig. 6). The fungal pathogen E22 was more sensitive in comparison with Ca10_13 when irenolone (**4**), anigorufone (**9**), isoanigorufone (**11**) and methoxyanigorufone (**7**) were assessed under dark conditions. Furthermore, these compounds displayed an enhanced antifungal activity when light conditions were applied to the experimental system (Fig. 6). Phenylphenalenones (Flors & Nonell 2006; Lazzaro *et al*. 2004) and perinaphthenones (Hidalgo *et al*. 2009) have been reported to be light‐dependent singlet oxygen producers, and these could significantly affect the fungal growth rate. Overall, the hypothesis that *M. fijiensis* strain Ca10_13 could tolerate higher concentrations of phenylphenalenones than the strain E22 was found to hold only for some compounds and under darkness.

Differences in the fungal growth rate between both *M. fijiensis* strains became more apparent when the incubation experiments were scaled up to almost 10 times the volume of liquid culture used for the microtitre bioassay. The growth rate of *M. fijiensis* strain E22 was much slower than that of strain Ca10_13 when phenylphenalenones in the range of 0.01 to 0.04 mg mL^−1^ were supplied in the incubation medium; hydroxyanigorufone (**5**) and 2‐phenyl‐1,8‐naphthalic anhydride (**3**) were the exceptions (Fig. S5). Therefore, the strain Ca10_13 was selected for further experiments. Because some plant pathogens have developed strategies to evade plant defences through a complex detoxification process (Barz & Welle 1992; Jeandet *et al*. 2010; Pedras & Taylor 1993), we wondered whether the resistance of *M. fijiensis* to some phenylphenalenones could be attributed to metabolic detoxification.

In fact, the strain Ca10_13 was able not only to metabolize most of the compounds into sulfate‐derivatives (compounds **5** and **9–12**; Table 2) but also to further degrade them into undetectable metabolites or even decompose them completely as in the case of hydroxyanigorufone (**5**) at the lowest concentration assessed. In general, the formation of sulfate conjugates through sulfotransferases constitutes a biochemical reaction, which is involved in such physiological processes as intracellular signalling, extracellular interactions and detoxification of xenobiotics (Chapman *et al*. 2004; Zhang *et al*. 1996). The present work is the first report of an inactivation or detoxification of phenylphenalenones by *M. fijiensis* through sulfate conjugation. In addition, to the best of our knowledge, ours is the first report of a detoxification reaction of any phenylphenalenone by a fungal organism. New strategies to control BLSD through inhibitors that target sulfotransferases in the microorganism may result from this new understanding of the role of sulfate conjugates.

Interestingly, only phenylphenalenones containing a hydroxyl group attached to C‐2 in the molecule were found to be sulfated (for hydroxyanigorufone, a sulfation in the free hydroxyl group at C‐4′ cannot be ruled out). Because conversion to sulfates seems to minimize the toxic effects of most phenylphenalenones, the fungal biomass production remained almost unaffected (Fig. S6). However, methoxyanigorufone (**7**), a metabolite which is missing a hydroxyl group (instead of the OH, an *O*‐methyl group is attached to C‐2), enormously affected growth and biomass production (Fig. S6). This supports the hypothesis that an α‐hydroxyketone feature helps the molecule to be recognized by the aryl sulfotransferase. In addition, the chemical pressure generated by compound (**7**) on the metabolism of the strain Ca10_13 was confirmed by the overproduction of soluble extracellular proteins (Fig. S7) and the inhibited production of ergosterol (Fig. S8). Because no metabolites derived from phenylphenalenones were found in the liquid culture medium during the incubation with the fungus, the overexpression of extracellular proteins observed in response to compounds **7** and **11** could not be associated with the external enzymatic detoxification of these metabolites. Instead, detoxification may suggest that these metabolites increase the physiological stress in the fungus, which then results in the overexpression of fungal proteins involved in the suppression of the plant defences (Dodds & Rathjen 2010; Escobar *et al*. 2015)*.* Identification of the proteins, which were overexpressed by the interaction with specific phenylphenalenones, would help explain the pathophysiological processes taking place during the interaction between *Musa* and *M. fijiensis*. Because of their role in the defence of *Musa* plants against *M. fijiensis*, methoxyanigorufone (**7**) and isoanigorufone (**11**) are considered the most relevant metabolites for further exploration of the molecular mode of phenylphenalenones.

## Supporting information


**Figure S1.** Chemical structures of the metabolites identified by 1D‐ and 2D NMR analysis in ‘Williams’ and ‘KTR’ *Musa* varieties.
**Figure S2.** Partial ^1^H NMR spectra showing the aromatic signals of dopamine and dopaol‐β‐D‐glucoside in a: infected leaf areas (A, see Fig. 2); b: non‐infected areas from (D); c: control *Musa* ‘Williams’ variety.
**Figure S3.** Fungal growth (data are normalized to the average growth of untreated control cultures) of *M. fijiensis* strain Ca10_13 after treatment with different concentrations of dopamine and dopaol‐β‐D‐glucoside.
**Figure S4.** Total content of phenylphenalenone‐type compounds (PP) produced by the Musa varieties ‘Williams’ and ‘KTR’ during 25 and 50 dpi with two different *M. fijiensis* strains. Letters a ‐ d indicate significant differences among treatments (One‐Way ANOVA, Holm‐Sidak *post hoc* test: P<0.001).
**Figure S5.** Data obtained from incubation of *M. fijiensis* strain E22 with different doses (0.01, 0.02 and 0.04 mg mL^−1^) of phenylphenalenones for 8 d.
**Figure S6.** Biomass produced by *M. fijiensis* strain Ca10_13 under *in vitro* treatment with different phenylphenalenone‐type compounds assessed at 0.01, 0.02 and 0.04 mg mL^−1^ for 8 d.
**Figure S7.** Soluble extracellular protein in *in vitro* culture medium of M. *fijiensis* strain Ca10_13 after incubation with phenylphenalenone‐type compounds at 0.01, 0.02 and 0.04 mg mL^−1^.
**Figure S8.** Ergosterol production by M. *fijiensis* strain Ca10_13 after *in vitro* incubationwith different phenylphenalenone‐type compounds at 0.01, 0.02 and 0.04 mg mL^−1^.
**Figure S9.** LC‐ESIMS analysis of anigorufone (9) and its metabolite in themethanolic extract of *M. fijiensis* Ca10_13mycelium. Panel **a**:UVchromatogramat 254 nm displaying peaks of anigorufone (*R*_t_ = 15.3 min) and an anigorufone‐derived metabolite (*R*_t_ = 7.8 min) identified as anigorufone‐2‐sulfate. Panel **b**: Extracted ion chromatogram (EIC) at *m/z* 353.2 [M +1]+, the mass corresponding to anigorufone‐2‐sulfate; Panel **c**: Mass spectrum of anigorufone‐2‐sulfate obtained at *R*_t_ = 7.8 min showing the molecular ion peak *m/z* 353.2 [M+H]+ and the fragment ion peak of *m/z* 273.2, corresponding to anigorufone (**9**).
**Table S1.** Black leaf streak disease symptoms developed by both *M. fijiensis* strains (E22 and Ca10_13) in ‘Williams’ and ‘KTR’ *Musa* varieties.
**Table S2.** Metabolites identified by 1D‐ and 2D‐NMR analysis in *Musa* varieties ‘Williams’ and ‘KTR’ and their up‐ and downregulation after infection with *M. fijiensis* E22 as inferred from the corresponding loading plots.

Supporting Information
