# Supplementary material for: Phenylphenalenones protect banana plants from infection by Mycosphaerella fijiensis and are deactivated by metabolic conversion
Source: Plant Cell Environ. 2015 Dec 11;39(3):492–513. doi: 10.1111/pce.12630 (PMC6220935; doi:10.1111/pce.12630)
Supplement: Supplementary file 1 — Supporting Information [file PCE-39-492-s001.docx]

**Phenylphenalenones protect banana plants from infection by *Mycosphaerella fijiensis* and are deactivated by metabolic conversion**

William Hidalgo^1^, Jima N. Chandran^1^, Riya Menezes^1^, Felipe Otálvaro^2^ and Bernd Schneider^1^*

^1^Max-Planck Institut für Chemische Ökologie, Beutenberg Campus, Hans-Knöll-Strasse 8, 07745, Jena, Germany

^2^Instituto de Química-Universidad de Antioquia, Calle 67# 53-108, A.A. 1226, Medellín, Colombia

**Supplementary information**

Fig. S1 p. 2

Fig. S2 p. 3

Fig. S3 p. 4

Fig. S4 p. 5

Fig. S5 p. 6

Fig. S6 p. 7

Fig. S7 p. 8

Fig. S8 p. 9

Fig. S9 p. 10

Table S1 p. 11

Table S2 p. 13


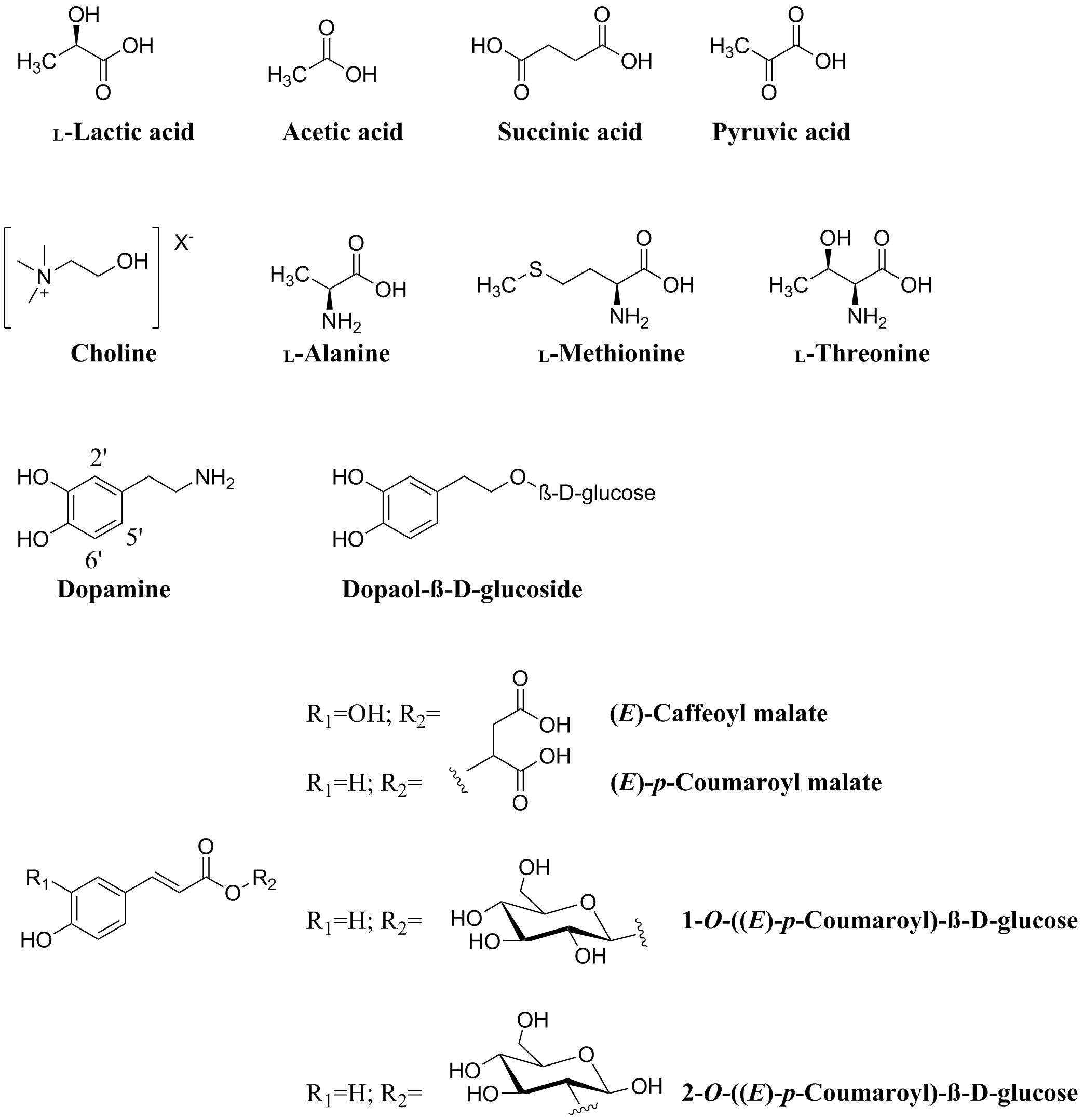


Fig. S1. Chemical structures of the metabolites identified by 1D- and 2D NMR analysis in 'Williams' and 'KTR' *Musa* varieties.


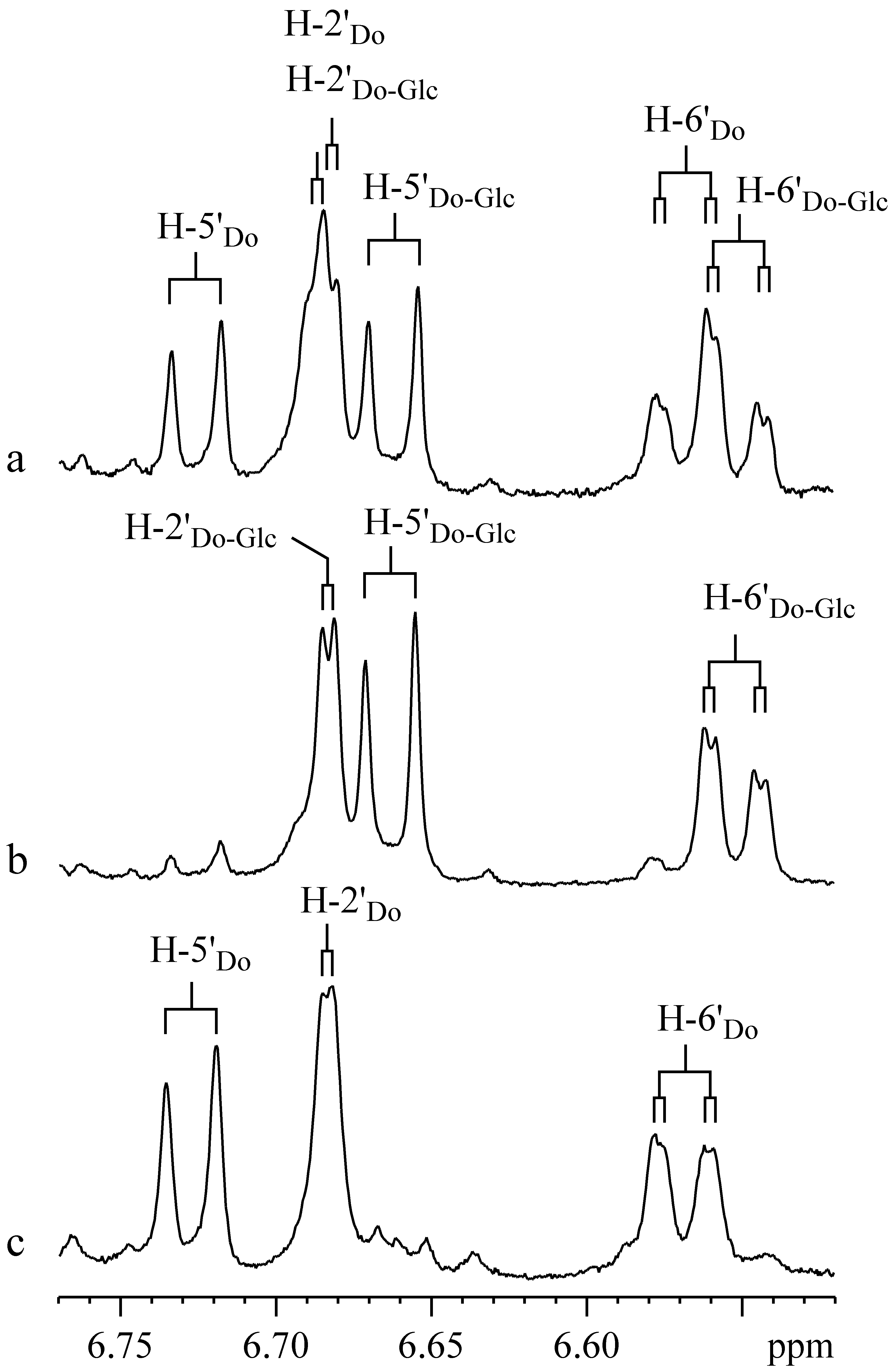


Fig. S2. Partial ^1^H NMR spectra showing the aromatic signals of dopamine and dopaol-β-d-glucoside in **a**: infected leaf areas (A, see Fig. 2); **b**: non-infected areas from (D); **c**: control *Musa '*Williams' variety. ^1^H NMR spectra of leaf areas (B) and (E) (not shown) resembled those of (D). Similar results were observable for the 'KTR' variety. Do: dopamine; Do-Glc: dopaol-ß-d-glucoside.


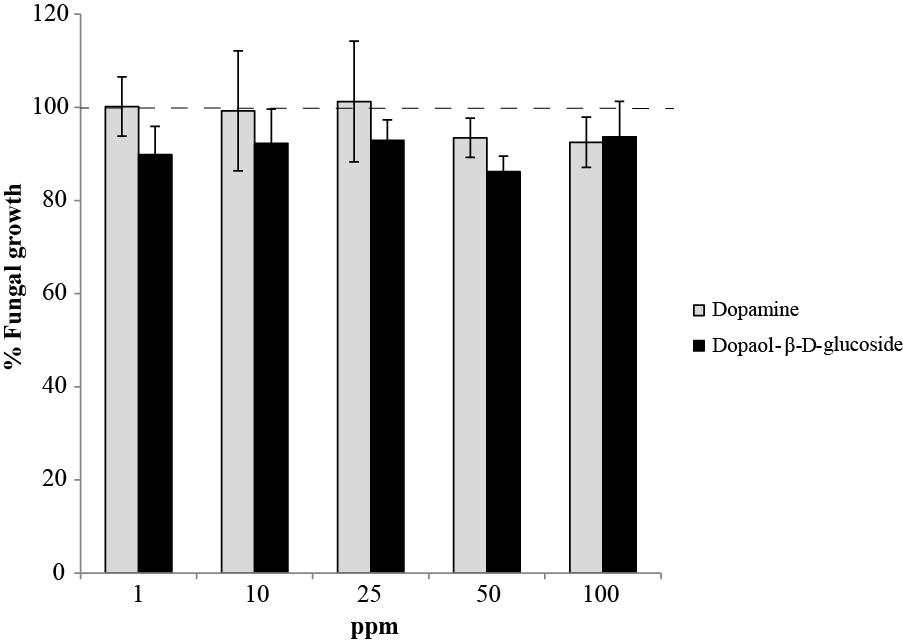


Fig. S3. Fungal growth (data are normalized to the average growth of untreated control cultures) of *M. fijiensis* strain Ca10_13 after treatment with different concentrations of dopamine and dopaol-β-d-glucoside.


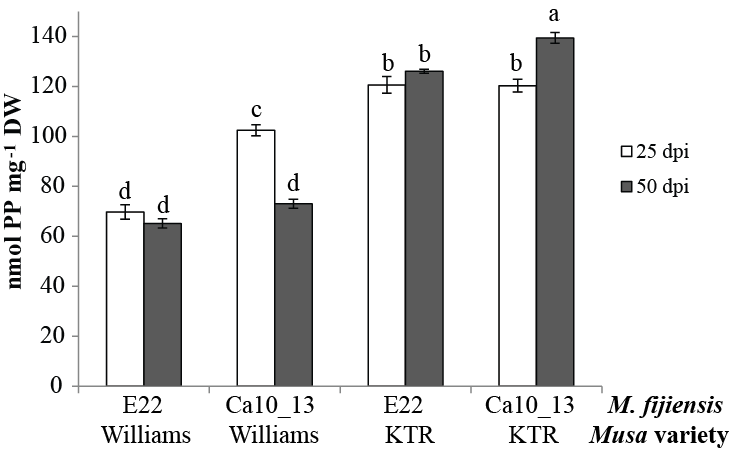


Fig. S4. Total content of phenylphenalenone-type compounds (PP) produced by the *Musa* varieties 'Williams' and 'KTR’ during 25 and 50 dpi with two different *M. fijiensis* strains. Letters a - d indicate significant differences among treatments (One-Way ANOVA, Holm-Sidak *post hoc* test: *P*<0.001).


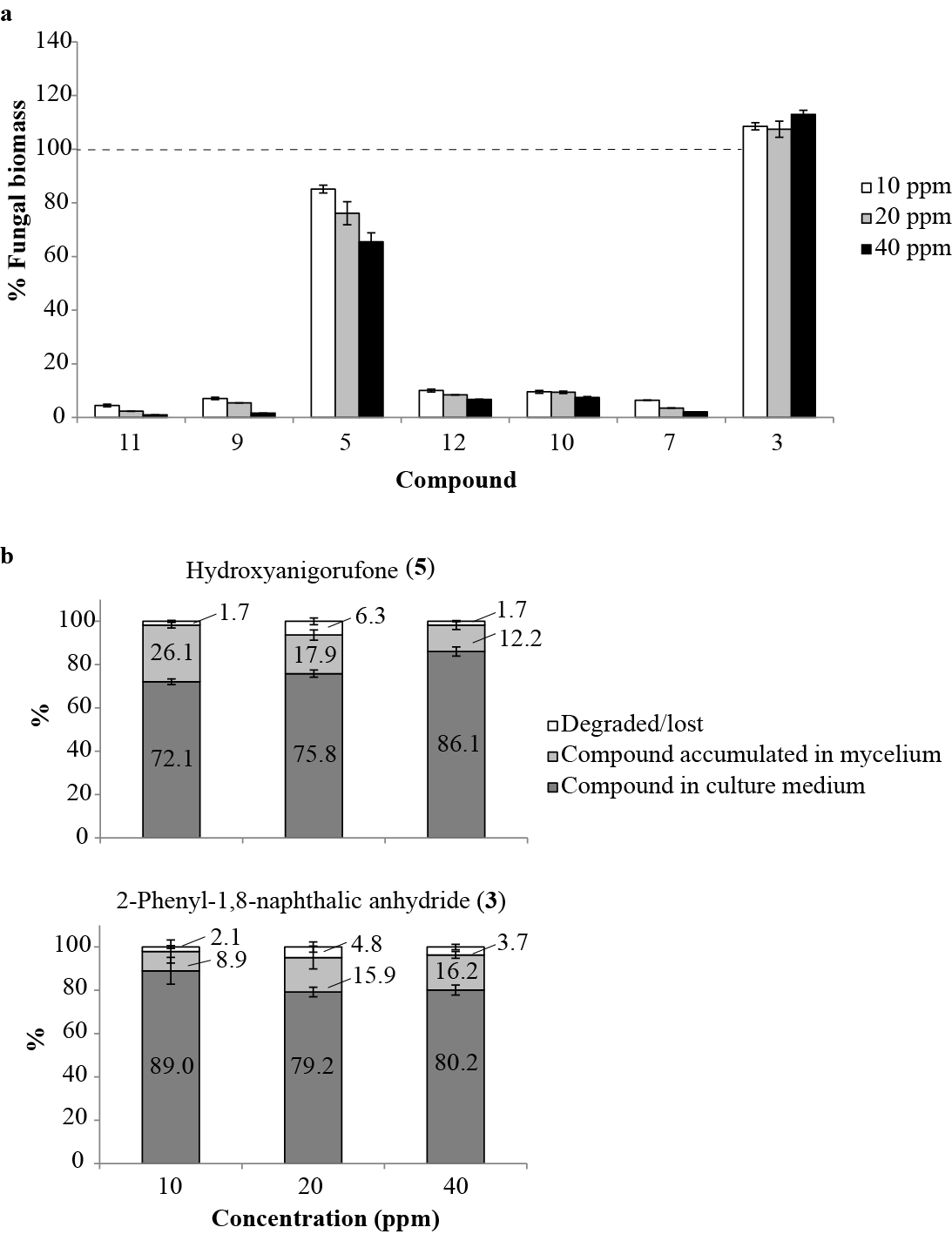


Fig. S5. Data obtained from incubation of *M. fijiensis* strain E22 with different doses (10, 20 and 40 ppm) of phenylphenalenones for 8 d. For chemical structures of compounds **3**, **5**, **7**, and **9** – **11**, see Table 1. Panel **a**. Fungal biomass produced under *in vitro* treatment with phenylphenalenones **3**, **5**, **7**, and **9** – **11**. Data were normalized for final biomass determination based on the growth of the non-treated fungus (control). Panel **b**. Quantification of the compounds **3** and **5** in the mycelium and the culture medium after incubation with the specified compound. Data were normalized as described in the legend of Fig. 7.

Fig. S6. Biomass produced by *M. fijiensis* strain Ca10_13 under *in vitro* treatment with different phenylphenalenone-type compounds assessed at 10, 20 and 40 ppm for 8 d. Data were normalized for final biomass determination based on the growth of the non-treated fungus (control). Letters a - b indicate significant differences among treatments with the specified compound (Two-Way ANOVA, Holm-Sidak *post hoc* test: *P*<0.001; n.s: not significant). For chemical structures of compounds **3**, **5**, **7**, and **9** – **11**, see Table 1.


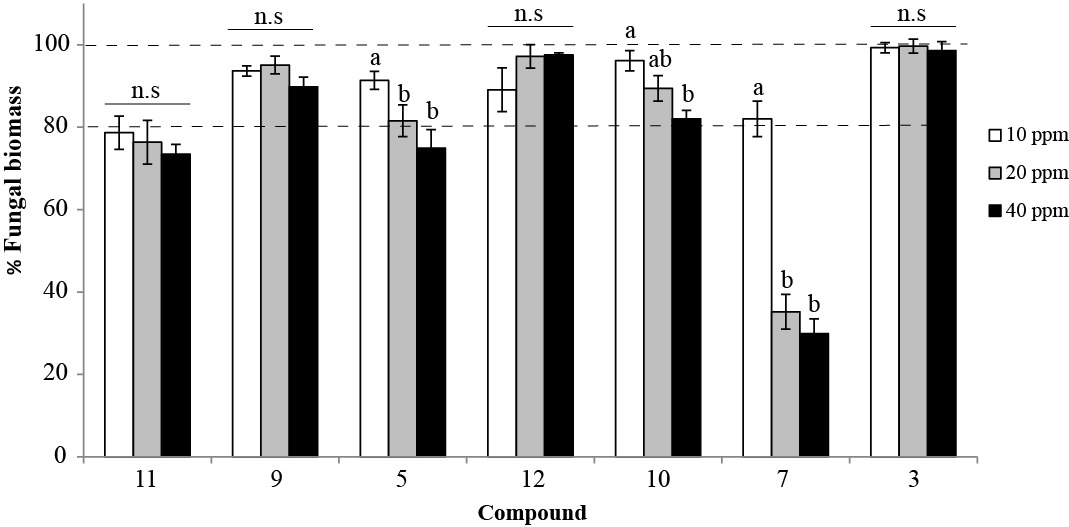

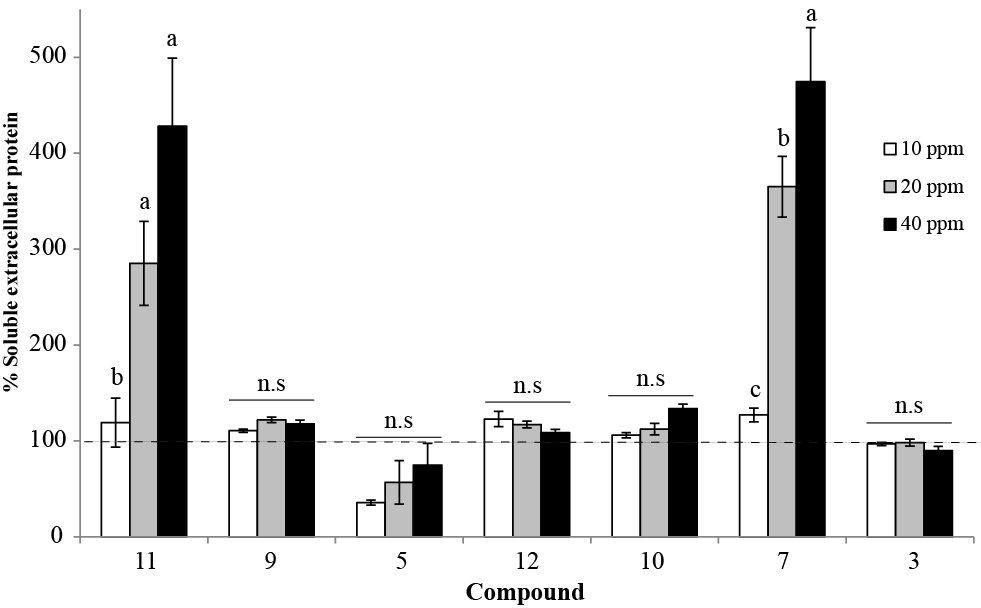


Fig. S7. Soluble extracellular protein in *in vitro* culture medium of *M. fijiensis* strain Ca10_13 after incubation with phenylphenalenone-type compounds at 10, 20 and 40 ppm. Data were normalized based on the total soluble extracellular protein determined to the non-treated fungal incubation (control). Letters a - c indicate significant differences among treatments with the specified compound (Krustal-Wallis One-way ANOVA on Ranks, Tukey test: *P*<0.05; n.s: not significant). For chemical structures of compounds **3**, **5**, **7**, and **9** – **11**, see Table 1.


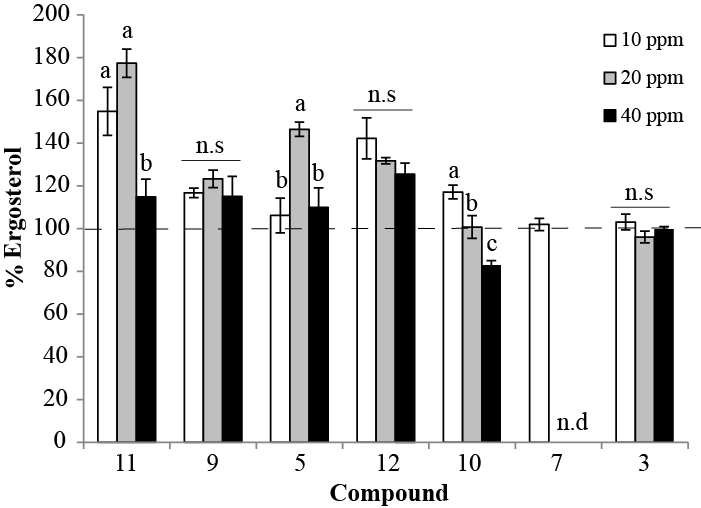


Fig. S8. Ergosterol production by *M. fijiensis* strain Ca10_13 after *in vitro* incubation with different phenylphenalenone-type compounds at 10, 20 and 40 ppm. Data were normalized based on the total ergosterol production determined for the non-treated fungal culture (control without phenylphenalenones). Letters a - c indicate significant differences among treatment (concentration) with the specified compound (Two-Way ANOVA, Holm-Sidak *post hoc* test: *P*<0.001; n.s: not significant). n.d: not detected. For chemical structures of compounds **3**, **5**, **7**, and **9** – **11**, see Table 1.


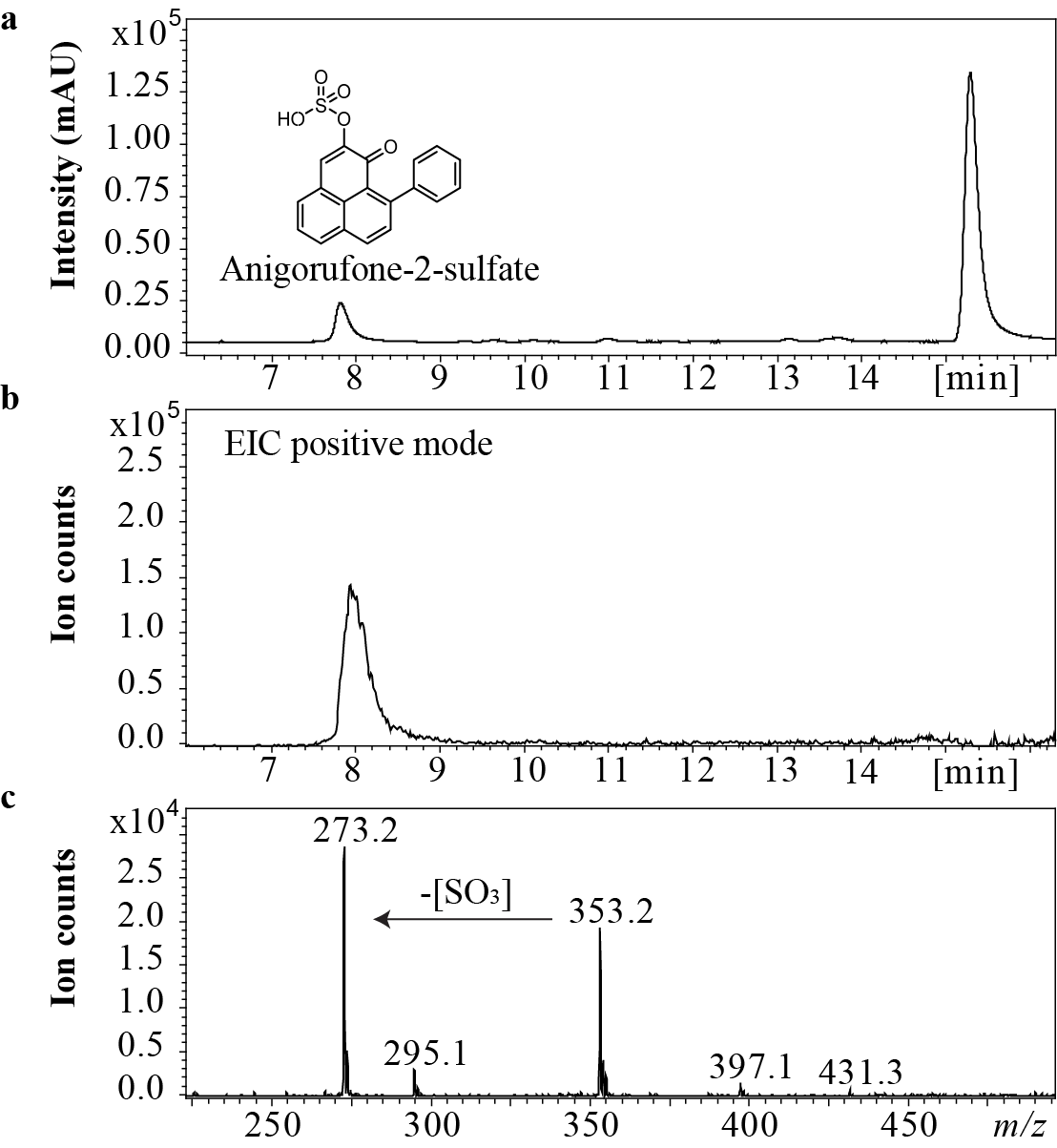


Fig. S9. LC-ESIMS analysis of anigorufone (**9**) and its metabolite in the methanolic extract of *M. fijiensis* Ca10_13 mycelium. Panel **a**: UV chromatogram at 254 nm displaying peaks of anigorufone (*R*_t_ = 15.3 min) and an anigorufone-derived metabolite (*R*_t_ = 7.8 min) identified as anigorufone-2-sulfate. Panel **b**: Extracted ion chromatogram (EIC) at *m/z* 353.2 [M+1]^+^, the mass corresponding to anigorufone-2-sulfate; Panel **c**: Mass spectrum of anigorufone-2-sulfate obtained at *R*_t_ = 7.8 min showing the molecular ion peak *m/z* 353.2 [M+H]^+^ and the fragment ion peak of *m/z* 273.2, corresponding to anigorufone (**9**).

Table S1. Black leaf streak disease symptoms developed by both *M. fijiensis* strains (E22 and Ca10_13) in 'Williams' and 'KTR' *Musa* varieties.

| Symptoms of Black Leaf Streak Disease (BLSD) caused by *Mycosphaerella fijiensis* | | | | |
| --- | --- | --- | --- | --- |
| Time (days) | Strain E22 | | Strain Ca10_13 | |
|  | *Musa* variety 'Williams' | *Musa* variety 'KTR' | *Musa* variety  'Williams' | *Musa* variety 'KTR' |
| 8 | 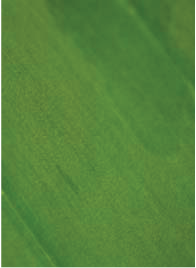 No symptoms | 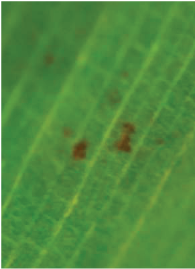 Red specks (0.5-1.0 mm diam.) on the abaxial side of the leaf | 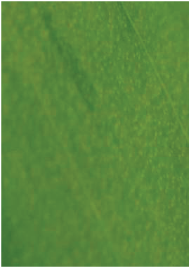 No symptoms | 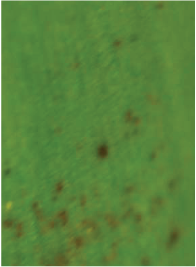 Reddish brown specks (0.5-2.0 mm diam.) on the abaxial side of the leaf |
| 16 | 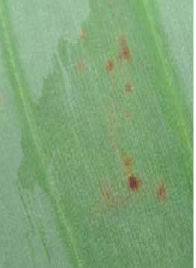 Reddish brown specks (less than 0.5 mm diam.) on the abaxial side of the leaf | 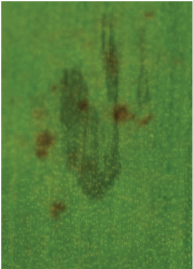 Small reddish brown spots (1-2 mm diam.) on the adaxial side of the leaf | 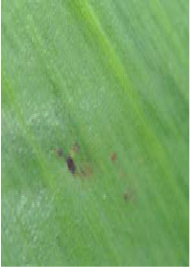 Brown dark spots (1-2 mm diam.) on the abaxial side of the leaf | 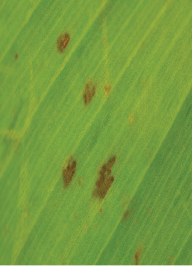 Reddish brown spots (1-3 mm diam.) on the abaxial side of the leaf |
| 25 | 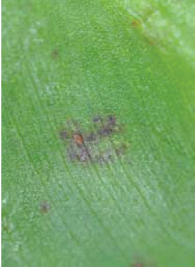 Brown and black blotches (3-5 mm diam.) on the adaxial side of the leaf | 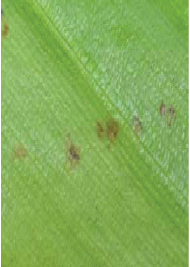 Brown dark spots (2-3 mm diam.) on the adaxial side of the leaf | 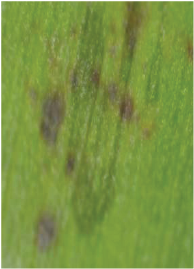 Brown and black spots (2-5 mm diam.) on the abaxial side of the leaf | 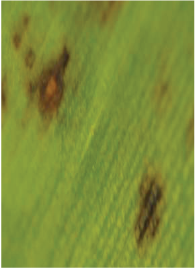 Reddish and brown dark spots (3-6 mm diam.) on the adaxial side of the leaf |
| 50 | 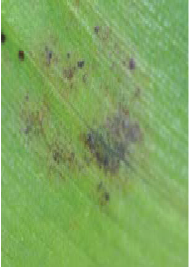 Asymmetric necrotic lesions (4-8 mm diam.) on the adaxial side of the leaf | 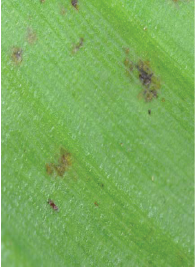 Reddish and brown spots (2-5 mm diam.) on the adaxial side of the leaf | 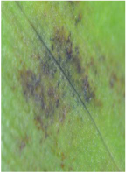 Necrotic lesions (10-12 mm diam.) on the adaxial side of the leaf | 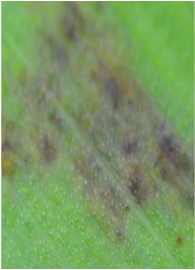 Necrotic lesions (10-15 mm diam.) on the adaxial side of the leaf |

.

Table S2. Metabolites identified by 1D- and 2D-NMR analysis in *Musa* varieties 'Williams' and 'KTR' and their up- and down-regulation after infection with *M. fijiensis* E22 as inferred from the corresponding loading plots.

| Entry | Metabolite^e^ | Position H/C | NMR data | | Assigned with | Changes after infection with *M. fijiensis* E22 in *Musa*:^d^ | |
| --- | --- | --- | --- | --- | --- | --- | --- |
|  |  |  | δ ^1^H (mult., *J* [Hz]) | δ ^13^C |  | 'Williams' | 'KTR' |
| 1 | Fatty acids | -CH_3_  -CH_3_ | 0.88 (t, 7.5 )  0.95 (t, 7.5) | 13.1  13.3 | HSQC | **↓** | ↓ |
| 2 | Lactic acid | -CH_3_  H/C-2 | 1.29^c^  4.09 | 22.3  71.8 | HSQC, HMBC | nd | nd |
| 3 | Acetic acid | -CH_3_  -COOH | 1.93 (s) | 25.9  175.9 | HSQC, HMBC | **↓** | nd |
| 4 | Succinic acid | H/C-2, 3  -COOH | 2.59 (s) | 31.6  177.0 | HSQC, HMBC | ↑ | ↑ |
| 5 | Pyruvic acid | - CH_3_  -COOH (α)  -COOH (β) | 2.35 (s) | 29.5  180.3  203.7 | HSQC, HMBC | **↓** | ↓ |
| 6 | Choline | H/C-1  H/C-2  -N^+^(CH_3_)_3_ | 3.5 (dd, 3.3, 5.0)  4.07 (m)  3.2 (s) | 73.1  58.3  53.2 | HSQC, HMBC | **↓** | ↓ |
| 7 | Glucose | CH-1 | 5.1 (d, 3.8) | 92.3 | HSQC, TOCSY | ↑ | ↑ |
| 8 | Sucrose | CH-1  CH-1' | 5.39 (d, 3.7)  4.09 (d, 8.5) | 92.3  77.7 | HSQC, TOCSY | ↑ | ↑ |
| 9 | l-Alanine^a^ | -CH_3_  H/C-2 | 1.46 (d, 7.3)  3.59^c^ | 15.9  54.6 | HSQC, COSY | **↓** | ↓ |
| 10 | l-Methionine^a^ | H/C-2  H/C-4  H/C-3  -CH_3_ | 3.81^c^  2.63 (dd, 7.8, 7.9)  2.19^c^  2.09^c^ | 55.0  32.5  31.6  17.9 | HSQC, COSY | ↑ | ↑ |
| 11 | l-Threonine^a^ | -CH_3_  H/C-2  H/C-3  -COOH | 1.3 (s)  3.55 (d, 3.5)  4.26^c^ | 22.2  60.7  68.6  173.6 | HSQC, HMBC | **↓** | ↓ |
| 12 | Dopamine^a^ | H/C-5'  H/C-2'  H/C-6' | 6.72 (d, 8.0)  6.69 (d, 1.9)  6.56 (d, 7.9) | 115.2  115.3  119.5 | HSQC | **↓** | **↓** |
| 13 | Dopaol-β-d-glucoside^b^ | H/C-2'  H/C-5'  H/C-6'  H/C-8'a | 6.68 (d, 1.9)  6.65 (d, 7.9)  6.54 (dd, 1.9. 7.9)  3.17 (dd, 8.9, 9.1) | 115.7  114.6  119.6  73.6 | HSQC, HMBC | ↑ | ↑ |
| 14 | (*E*)-Caffeoyl malate | H/C-2  H/C-3  H/C-5'  H/C-6'  H/C-7'  H/C-8' | 5.32^c^  2.94 (dd, 3.1, 15.9) 2.83 (dd, 8.3, 15.5)  6.79 (d, 8.0)  6.95 (d, 2.0, 8.2)  7.57 (d, 16.0)  6.29 (d, 16.0) | 71.9  39.6  115.1  121.5  145.4  114.1 | HSQC, HMBC, TOCSY | nd | nd |
| 15 | (*E*)-*p*-Coumaroyl malate | H/C-2  H/C-3  H/C-2'/6'  H/C-3'/5'  H/C-7' | 5.32^c^  2.94 (dd, 3.1, 16.0) 2.83 (dd, 8.3, 16.0)  7.47 (d, 8.8)  6.8 (d, 8.9)  7.65 (d, 16.0) | 71.9  39.6  131.2  145.6 | HSQC, HMBC, TOCSY | nd | nd |
| 16 | 1-*O*-((*E)*-*p*-Coumaroyl)-β-d-glucose | H/C-3'/5’  H/C-2'/6’  H/C-7’  H/C-8’ | 6.8 (d, 8.6)  7.52 (d, 8.6)  7.73 (d, 16.0)  6.44 (d, 16.0) | 115.3  130.2  148.8  112.7 | HSQC, HMBC, TOCSY, COSY | **↓** | **↓** |
| 17 | 2-*O*-((*E*)-*p*-Coumaroyl)-ß-d-glucose | H/C-7’  H/C-8’ | 7.62 (d, 16.0)  6.34 (d, 16.0) | 145.5  114.2 | HSQC, HMBC | nd | nd |
| 18 | Irenolone | H/C-3'/5'  H/C-2'/6' | 6.96 (d, 8.1)  7.34 (d, 8.4) | 115.4  131.5 | HSQC, HMBC | ↑ | ↑ |
| 19 | Hydroxyanigorufone | H/C-2'/6'  H/C-3'/5'  H/C-7 | 7.23 (d, 8.5)  6.85 (d, 8.5)  8.27 (d, 8.4) | 130.6  115.5  136.1 | HSQC, HMBC | ↑ | ↑ |

^a^ Metabolites identified by comparing NMR data with authentic standards

^b^ Metabolite isolated and 1D- and 2D-NMR data are matched with the data reported in the literature (Franzyk *et al.* 2004)

^c^ Signals or multiplicities were not determined because of signal overlap.

^d^ (↑) or (↓): up- or down-regulation of metabolites.

^e^ Chemical structures are shown in Fig. S1
